# Epigenetics of Male Infertility: The Role of DNA Methylation

**DOI:** 10.3389/fcell.2021.689624

**Published:** 2021-07-22

**Authors:** John Charles Rotondo, Carmen Lanzillotti, Chiara Mazziotta, Mauro Tognon, Fernanda Martini

**Affiliations:** Laboratories of Cell Biology and Molecular Genetics, Department of Medical Sciences, University of Ferrara, Ferrara, Italy

**Keywords:** DNA methylation, sperm DNA, male infertility, imprinting, infertility, epigenomics, sperm, imprinted genes

## Abstract

In recent years, a number of studies focused on the role of epigenetics, including DNA methylation, in spermatogenesis and male infertility. We aimed to provide an overview of the knowledge concerning the gene and genome methylation and its regulation during spermatogenesis, specifically in the context of male infertility etiopathogenesis. Overall, the findings support the hypothesis that sperm DNA methylation is associated with sperm alterations and infertility. Several genes have been found to be differentially methylated in relation to impaired spermatogenesis and/or reproductive dysfunction. Particularly, DNA methylation defects of MEST and H19 within imprinted genes and MTHFR within non-imprinted genes have been repeatedly linked with male infertility. A deep knowledge of sperm DNA methylation status in association with reduced reproductive potential could improve the development of novel diagnostic tools for this disease. Further studies are needed to better elucidate the mechanisms affecting methylation in sperm and their impact on male infertility.

## Introduction

Male infertility affects about 15% of couples worldwide (Agarwal et al., 2015). In reproductive age, approximately 7% of males suffer from infertility (Cooper et al., 2009; Krausz, 2011; Rotondo et al., 2013). Male infertility is a multifactorial disease comprising a wide variety of disorders (Abrao et al., 2013; Stouffs et al., 2014; Contini et al., 2018; Tognon et al., 2020). Endocrine and immunological disorders, anatomical and genetic abnormalities as well as infections of the genital tract can affect the male reproductive potential (Abrao et al., 2013; Stouffs et al., 2014). Several other factors including age, stress, and lifestyle, such as obesity, smoking, and alcohol, have been associated with male infertility (Kovac et al., 2015; Craig et al., 2017; Ilacqua et al., 2018).

In recent years, a number of studies have focused on the role of epigenetics in spermatogenesis and male infertility (Seisenberger et al., 2012; Laurentino et al., 2015; Stuppia et al., 2015; Urdinguio et al., 2015; Laqqan et al., 2017b; Nasri et al., 2017; McSwiggin and O’Doherty, 2018). Epigenetics refers to the gene regulation process without changes in DNA sequence and includes DNA methylation, posttranslational histone modifications and microRNA (miRNA) regulation (Giacone et al., 2019; Stomper et al., 2021). Several specific epigenomic/epigenetic modifications are established during spermatogenesis to form highly specialized mature sperm cells, allowing significant reorganizations of sperm chromatin structure (Jenkins and Carrell, 2011). Therefore, spermatogenesis is particularly vulnerable to epigenetic alterations. Dysregulations in the DNA methylation process during spermatogenesis can result in the abnormal expression of target genes, which may lead to infertility (Cho et al., 2003; Aston et al., 2012). While many epigenetic abnormalities causing male reproductive dysfunction are still unknown, it is likely that most cases of idiopathic infertility could be accounted for underlying DNA methylation mechanisms (Rose and Klose, 2014).

This review provides an overview on gene and genome methylation and its regulation during spermatogenesis and the current knowledge of those DNA methylation defects potentially involved in the etiopathogenesis of male infertility.

## Methods

We performed an investigation of the scientific literature by searching PubMed (Medline) database until March 2021. All studies investigating the relationship between DNA methylation and male infertility published from 1987 up to March 2021 were reviewed for specific topic areas, and the most relevant reports were included. The literature search was performed using the following keywords (alone and/or in combination): epigenomics, epigenetics, gene, methylation, DNA methylation, genome, methylome, hypermethylation, hypomethylation, regulation, genomic imprinting, imprinted genes, sperm, sperm cells, spermatozoa, semen parameters, spermatogenesis, infertility, subfertility, sterility, and male infertility. The reference lists of all publications included in this review have also been considered for additional relevant works.

## DNA Methylation in Germ Cell Development

**FIGURE 1:**
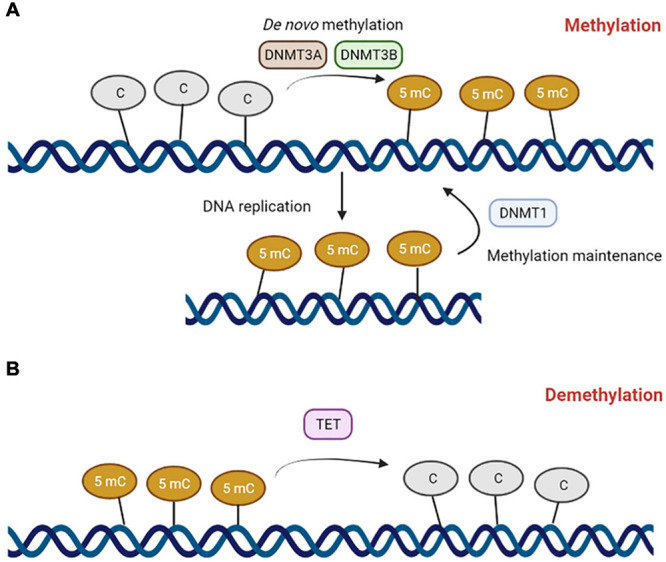
The DNA methylation machinery. **(A)** DNA methyltranferase 3A (DNMT3A) and 3B (DNMT3B) catalyze *de novo* methylation reactions on cytosines belonging to CpG dinucleotides to form 5-methylcytosine. DNA methyltransferase 1 (DNMT1) mediates DNA methylation maintenance. **(B)** Ten-eleven translocation (TET) enzymes mediates DNA demethylation reactions.

DNA methylation is a biochemical process where a nucleotide is enzymatically methylated with a methyl group (–CH_3_) at the five-carbon position, usually cytosine (Moore et al., 2013; Rotondo et al., 2021). Cytosine methylation predominantly occurs at the CpG dinucleotide (Figure 1). Regions rich in CpGs are called CpG islands (CGI) and are usually found in gene promoters, where gene expression is regulated through methylation (Greenberg and Bourc’his, 2019; Rotondo et al., 2020b). CpG methylation in the promoters of genes typically leads to gene silencing (Rotondo et al., 2016, 2018a). The suppression of gene expression can occur by DNA methylation itself that, in some cases, can prevent the binding of transcriptional factors (Moore et al., 2013; Yin et al., 2017). Methylated DNA could also be recognized and bound by the methyl-binding proteins methyl CpG binding protein 2 and methyl-CpG-binding domain protein-1, -2, and -4, which recruit the enzymes histone methyltransferases and histone deacetylases to trigger histone modifications and chromatin packing, leading to the repression of gene expression (Parry and Clarke, 2011).

**FIGURE 2:**
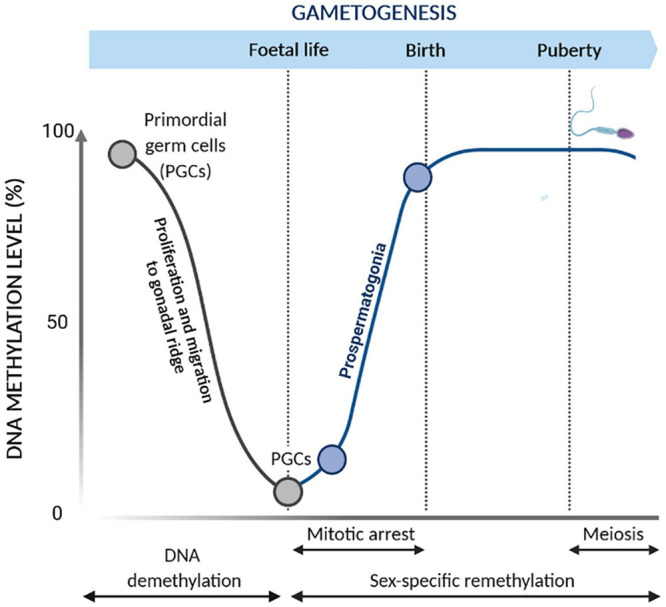
Epigenetic reprogramming during gametogenesis. Changes of DNA methylation occur during primordial germ cell (PGC) development. During the proliferation and migration into the gonadal ridge, PGCs undergo global demethylation to remove parental imprints. Subsequently, reestablishment of the male germ cell DNA methylation patterns occurs during gametogenesis. *De novo* methylation occurs prior to meiosis in mitotically arrested cells while being completed before birth.

DNA methylation plays a pivotal role in regulating germ cell development (Reik and Walter, 2001; Castillo et al., 2018; Hanna et al., 2018; Lobo et al., 2019; Sharma et al., 2019; Wasserzug-Pash and Klutstein, 2019). During germ cell development, the genome is widely remodeled by waves of DNA demethylation and methylation (Figure 2; Reik and Walter, 2001; Li, 2002; Santos and Dean, 2004; Rousseaux et al., 2005; Biermann and Steger, 2007; Marcho et al., 2019). The wave of DNA demethylation occurs in the primordial germ cells (PGCs), which are precursors of male and female germ cells. Initially, the content of DNA methylation is equally distributed between PGCs and embryo somatic cells. Upon migration of PGCs from the epiblast to the gonadal ridge, the DNA methylation marks are extensively erased (Monk et al., 1987; Santos and Dean, 2004), resulting in lower methylation levels in PGCs compared to those in embryo somatic cells (Gkountela et al., 2015; Guo et al., 2015; Tang et al., 2015; Von Meyenn and Reik, 2015). These epigenetic modifications are essential for the genome reprogramming of PGCs, allowing sex-specific germ cell development during embryo development (Reik and Walter, 2001; Yao et al., 2015). *De novo* methylation proceeds in prospermatogonia or gonocytes arrested in mitosis, and it is firstly established at the repeated DNA sequences, such as retrotransposons, and then at the imprinted genes, leading to appropriate sex-specific methylation patterns in the germ cells.

**FIGURE 3:**
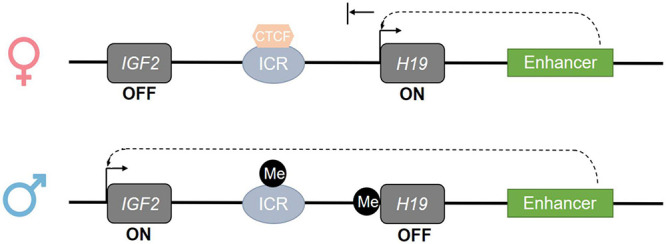
Epigenetic regulation of the imprinted *IGF2-H19* gene cluster. The regulation of the imprinted genes is mediated by imprinting control regions (ICR) whose methylation regulates the imprinted genes located at the same gene cluster. On the maternal allele, at the *IGF2-H19* locus, the ICR is de-methylated; this allows CTCF binding and promotes the expression of *H19* and silencing of *IGF2* through down-stream enhancer activity. On the paternal allele, at the *IGF2-H19* locus, the ICR and *H19* are methylated; this prevents CTCF binding and induces the inactivation of *H19* and the expression of *IGF2.* Consistently, *IGF2* is expressed in the sperm cell (Cannarella et al., 2019).

DNA methylation establishment in the germline is of particular importance, as failure to establish correct methylation in retrotransposons and imprinted genes has serious consequences for embryo development. Indeed, retrotransposons, such as *long interspersed nuclear elements 1* (*LINE1*), are interspersed repeated DNA sequences which are able to propagate throughout the genome. Lack of methylation in retrotransposons could allow their propagation throughout the genome, causing insertional mutagenesis and, in turn, several diseases, including male infertility (Solyom and Kazazian, 2012). Regarding the imprinted genes, methylation regulates the expression through an important process termed “genomic imprinting,” which leads to the expression of either the maternal or paternal allele. When maternal/paternal alleles undergo global demethylation upon fertilization, the imprinted genes preserve the methyl marks of the parental genome, leading to parental-origin patterns of mono-allelic gene expression. This process occurs in specific sequences called differentially methylated regions (DMRs), also known as imprinting control regions (ICRs) (Habib et al., 2019). For instance, the imprinted genes *IGF2-H19* share common enhancers and ICR located downstream and upstream of the *H19* gene, respectively. Under normal circumstances, ICR and *H19* are methylated in sperm cells and unmethylated in oocytes, leading to the reciprocal expression of the maternal *H19* allele and paternal *IGF2* allele in somatic cells (Figure 3; Peters, 2000; Lanzillotti et al., 2021). Other paternally imprinted genes have been found to be similarly regulated in sperm cells, such as *Ras Protein Specific Guanine Nucleotide Releasing Factor 1*, *Delta Like Non-Canonical Notch Ligand 1*, and *Zinc Finger DBF-Type Containing 2* (Arnaud, 2010; Gunes et al., 2016). Contrariwise, maternally imprinted genes are methylated in female germ cells, while they are expressed in male germ cells, including *mesoderm-specific transcript* (*MEST*), also known as *paternally expressed gene 1* (*PEG1*), *paternally expressed 3* (*PEG3*), and *small nuclear ribonucleoprotein polypeptide N* (*SNRPN*) (Mayer et al., 2000; Higashimoto et al., 2003; Broad et al., 2009; Hammoud et al., 2010; Zheng et al., 2011; Zhao et al., 2015; Bruno et al., 2018). However, failure in the maintenance of imprinted gene methylation patterns in the germline might occur, and this has been associated with low sperm quality and pregnancy rate and impaired post-fertilization development (Rotondo et al., 2013; Kitamura et al., 2015; McSwiggin and O’Doherty, 2018).

DNA methylation is carefully coordinated by a family of enzymes named DNA methyltransferases (DNMTs). Four members of this family, DNMT1, DNMT3A, DNMT3B, and DNMT3C, are endowed with catalytic activity (Lopomo et al., 2016). DNMT1, also known as the maintenance DNMT, copies pre-existing methylation marks onto the new strands following DNA replication (Baylin and Jones, 2011). DNMT3A and DNMT3B are *de novo* methyltransferases, as they are able to methylate previously unmethylated DNA sequences (Baylin and Jones, 2011). DNMT3C, along with *P-element induced wimpy testis/interacting RNA* (*PIWI/piRNA*) (Yang and Wang, 2016; Nagamori et al., 2018), is responsible for the promoter methylation of retrotransposons in the male germline (Barau et al., 2016). piRNAs are small non-coding RNAs that form RNA–protein complexes through interaction with PIWI proteins, a gonad-specific class of Argonaute proteins (Yang and Wang, 2016). These RNA–protein complexes are essential for the *LINE1* methylation processes in male germ cells (Aravin et al., 2008; Kuramochi-Miyagawa et al., 2008). DNA methylation is a reversable chemical modification, and active demethylation processes are mediated by erasing DNA methylation mechanisms, mainly controlled by Ten-Eleven Translocation (TET) enzymes, such as TET1, -2, and -3, (Melamed et al., 2018). TET enzymes specifically convert 5mC to 5-hydroxymethylcytosine (5hmC). The TET-mediated oxidation of 5mC induces DNA demethylation by passive or active processing. As 5hmC is not recognized by DNMT1 during DNA replication, passive DNA demethylation can occur in proliferating cells (Valinluck and Sowers, 2007). In the active process, TETs convert 5hmC to 5-formylcytosine (5fC) and then 5fC into 5-carboxylcytosine (5cC). 5fC and 5cC are both recognized and excised by the base-excision DNA repair machinery and subsequently replaced with cytosine (Williams et al., 2012; Jin et al., 2015).

## DNA Methylation and Male Infertility

### Overview

DNA methylation plays a critical role during spermatogenesis (Gunes et al., 2018b; Fend-Guella et al., 2019; Franzago et al., 2019; Huang et al., 2019; Pisarska et al., 2019). The correct methylation of DNA ensures proper chromatin condensation in the sperm head, enabling sperm maturation and its ability in fertilization and post-fertilization events (Carrell, 2019). Contrariwise, incomplete or abnormal condensation of the sperm chromatin results in damaged DNA, leading to the impairment of egg cell fertilization and/or reduction in pregnancy rates (Benchaib et al., 2005; Miller et al., 2009; McSwiggin and O’Doherty, 2018). Considering these aspects, several studies have analyzed the gene and genome methylation levels of sperm DNA in association with male reproductive dysfunctions (Houshdaran et al., 2007; Rotondo et al., 2012, 2013; Das et al., 2017; Alkhaled et al., 2018). These studies have mainly, but not only, investigated associations between improper DNA methylation in spermatozoa genome and negative variations in semen parameters, such as concentration (oligozoospermia), morphology (teratozoospermia), and progressive motility (asthenozoospermia), alone or in combinations, i.e., oligoasthenozoospermia (OA), oligoteratozoospermia, asthenoteratozoospermia (AT), and oligoasthenoteratozoospermia (OAT). Semen samples are considered normal when the concentration, motility, and morphology are ≥15 × 10^6^ sperm/ml, ≥32% (sperm progressive motility), and ≥4% normal, respectively (Cooper et al., 2009).

### Gene Methylation Changes

Several genes carrying defective methylation have been associated with abnormalities in semen parameters (Table 1). One of the most highly studied genes is *methylenetetrahydrofolate reductase* (*MTHFR*) (Haggarty, 2015; Saraswathy et al., 2018; Coppedè et al., 2019; Mishra et al., 2019; Kulac et al., 2021), which is a key regulatory enzyme involved in folate metabolism and in DNA synthesis and methylation (Födinger et al., 2000). In male mice testes, the activity of MTHFR is five times higher than in other organs (Chen, 2001), whereas its inactivation results in hyperhomocysteinemia, decreased methylation capacity, and the arrest of spermatogenesis due to sperm DNA hypomethylation (Chen, 2001). In humans, a number of studies have pointed out that impairment of the *MTHFR* gene can contribute to disease, including male infertility (Stangler Herodež et al., 2013; Tara et al., 2015; Coppedè et al., 2016, 2019; Lévesque et al., 2016; Asim et al., 2017). Mutations/polymorphisms in *MTHFR* gene are broadly known causes of reduced MTHFR enzyme activity in sperm cells, resulting in reduced methionine availability and decreased DNA methylation (Gupta et al., 2011; Montjean et al., 2011; Murphy et al., 2011; Safarinejad et al., 2011; Choi et al., 2016; Poorang et al., 2018; Ullah et al., 2019). The improper methylation of *MTHFR* gene in sperm germ cells may likewise result in decreased MTHFR enzyme activity, leading to aberrant methylation of sperm DNA. Hypermethylation of *MTHFR* has been found in testes from males affected by non-obstructive azoospermia and in sperm cells from males affected by oligozoospermia and teratozoospermia as well as in idiopathic infertile males (Khazamipour et al., 2009; Wu et al., 2010; Botezatu et al., 2014; Karaca et al., 2017). In our previous studies, hypermethylation at the *MTHFR* promoter has been found to be associated with aberrant concentration, motility, and morphology in males from infertile couples affected by recurrent spontaneous abortion (Rotondo et al., 2012). In addition, we have found a correlation between *MTHFR* gene promoter hypermethylation and extensive methylation defects at the paternal imprinted gene *H19* in sperm DNAs from infertile males with both normal and abnormal semen parameters. These results have suggested that *MTHFR* hypermethylation-induced inactivation may affect methylation at the imprinted loci of sperm cells, leading to impaired fertilization (Rotondo et al., 2013). Contrariwise, no correlation was found between methylation errors at *H19* and *MEST* genes and inactivating *MTHFR* C677T single-nucleotide polymorphism (SNP) [a cytosine (C) to a thymine (T) substitution at position 677] in sperm DNA from infertile patients with defective semen parameters. However, a higher incidence trend of aberrant DNA methylation among severe oligozoospermic infertile males carrying C677T genotype was observed (Louie et al., 2016).

**TABLE 1:** Aberrant methylation of non-imprinted genes associated with abnormalities of semen parameters.

**Gene**	**Function**	**Associated sperm parameters**	**References**
*ALU YB8*	Repetitive sequences	Normozoospermia	Urdinguio et al. (2015)
*BCAN*	Extracellular matrix formation	Oligo-/oligoasthenozoospermia	Sujit et al. (2018)
*CPEB2*	Tumor suppressor	Oligo-/oligoasthenozoospermia	Sujit et al. (2018)
*CREM*	Germline regulator	Not specified	Nanassy and Carrell (2011)
*CRISPLD1*	Cysteine-rich secretory proteins	Oligo-/oligoasthenozoospermia	Sujit et al. (2018)
*D4Z4*	Repetitive sequences	Normozoospermia	Urdinguio et al. (2015)
*DAZL*	Germline regulator and gametogenesis	Oligoasthenoteratozoospermia	Navarro-Costa et al. (2010)
*DNMT1*	DNA-methyltransferase	Non-obstructive azoospermia	Uysal et al. (2019)
*DNMT3A*	DNA-methyltransferase	Non-obstructive azoospermia	
*DNMT3B*	DNA-methyltransferase	Non-obstructive azoospermia	
*EZH2*	Histone methyltranferases	Oligo-/oligoasthenozoospermia	Sujit et al. (2018)
*HDAC4*	Histone deacetylases	Oligo-/oligoasthenozoospermia	
*HLA-C*	Antigen-presenting MHC I	Oligo-/oligoasthenozoospermia	
*HLA-DQA1*	Antigen-presenting MHC II	Oligo-/oligoasthenozoospermia	
*HLA-DRB6*	Antigen-presenting MHC II	Oligo-/oligoasthenozoospermia	
*HRAS*	GTPase	Oligoasthenoteratozoospermia	Houshdaran et al. (2007)
*HSPA1L*	Molecular chaperone	Normozoospermia	Jenkins et al. (2016b)
*HSPA1B*	Molecular chaperone	Normozoospermia	
*JMJD1C*	Histone demethylases	Oligo-/oligoasthenozoospermia	Sujit et al. (2018)
*KDM4C*	Histone demethylases	Oligo-/oligoasthenozoospermia	
*LINE1*	Repetitive sequences	Normozoospermia	Urdinguio et al. (2015)
		Oligoasthenoteratozoospermia	Boissonnas et al. (2010)
		Not specified	Khambata et al. (2021)
*LPHN3*	Member of GPCR	Oligo-/oligoasthenozoospermia	Sujit et al. (2018)
*MLH1*	DNA mismatch repair	Oligozoospermia	Gunes et al. (2018a)
		Oligoasthenoteratozoospermia	Hekim et al. (2021)
*MT1A*	Metallothionein	Oligoasthenoteratozoospermia	Houshdaran et al. (2007)
*MTHFR*	Methylation regulator	Non-obstructive azoospermia	Khazamipour et al. (2009)
		Normo-/oligozoospermia	Wu et al. (2010)
		Oligoasthenozoospermia	Botezatu et al. (2014)
		Oligozoospermia	Rotondo et al. (2012)
		Normozoospermia	Karaca et al. (2017)
		Non-obstructive azoospermia	Kulac et al. (2021)
		Oligozoospermia	
*NBL2*	Repetitive sequences	Normozoospermia	Urdinguio et al. (2015)
*NTF3*	Neurotrophic factor	Oligoasthenoteratozoospermia	Houshdaran et al. (2007)
*P16*	Tumor suppressor	Asthenozoospermia	Zhang et al. (2019)
*PAX8*	Transcription factor	Oligoasthenoteratozoospermia	Houshdaran et al. (2007)
PIWIL2	Endoribonuclease	Non-obstructive azoospermia/oligozoospermia	Heyn et al. (2012)
*RHOXF1*	Transcription factor	Oligo-/astheno/-teratozoospermia	Richardson et al. (2014)
*RHOXF2*	Transcription factor	Oligo-/astheno/-teratozoospermia	
*rs2656927*	SNP variants of *UHRF1*	Oligozoospermia	Zhu et al. (2019)
*rs8103849*	SNP variants of *UHRF1*	Oligozoospermia	
*SAT2CHRM1*	Spermine N1-acetyltransferase	Oligoasthenoteratozoospermia	Houshdaran et al. (2007)
*SFN*	Regulator of mitotic translation	Oligoasthenoteratozoospermia	
*SOX30*	Transcription factor	Non-obstructive azoospermia	Han et al. (2020)
*SPATA4*	Apoptosis regulator	Oligozoospermia	Sujit et al. (2020)
*SPATA5*	ATP-dependent chaperone	Oligozoospermia	
*SPATA6*	Myosin light chain binding	Oligozoospermia	
*SPATA7*	Microtubule cytoskeleton organization	Oligo-/oligoasthenozoospermia	Sujit et al. (2018)
*SPATA16*	Testis-specific protein	Oligo-/oligoasthenozoospermia	
*SPATA22*	Meiosis-specific protein	Oligo-/oligoasthenozoospermia	
*TDRD1*	Repressor of transposable elements	Non-obstructive azoospermia/oligozoospermia	Heyn et al. (2012)
*TET1*	Dioxygenase	Oligoasthenozoospermia	Ni et al. (2016)
*TET2*	Dioxygenase	Oligoasthenozoospermia	
*TET3*	Dioxygenase	Oligoasthenozoospermia	
*VDR*	Transcription factor	Oligo-/astheno/-teratozoospermia	Vladoiu et al. (2017)
*XRCC1*	Enzyme binding	Oligoasthenoteratozoospermia	Metin Mahmutoglu et al. (2021)

Germline mutations arise from replication errors, potentially resulting in reproductive health impairment (Gao et al., 2019; Altakroni et al., 2021). Accordingly, the genetic defects of DNA mismatch repair mechanisms have been related to impaired spermatogenesis and male infertility (Gunes et al., 2015; Hekim et al., 2021; Metin Mahmutoglu et al., 2021). A recent study has investigated the methylation profile of the DNA mismatch repair gene *MutL alpha* (*MLH1*) in sperm DNA from oligozoospermic patients and normozoospermic males enrolled as the control group (Gunes et al., 2018a). The results indicated an association between *MLH1* hypermethylation and male infertility, suggesting that this gene might be under epigenetic regulation during sperm cell development (Gunes et al., 2018a). High methylation levels of the DNA repair *X-ray repair cross-complementing protein 1* gene have been found in OAT males, thereby suggesting that the improper methylation of this gene may have a role in sperm chromatin condensation and OAT (Metin Mahmutoglu et al., 2021). Contrariwise, two additional well-known DNA repair genes, i.e., *breast cancer type 1 susceptibility protein* (*BRCA1*) and *BRCA2*, did not show defective methylation in infertile men with OAT (Kabartan et al., 2019).

Several other genes found aberrantly methylated in altered sperm samples from infertile males have been investigated at a single-study level (Table 1). Overall, several abnormally methylated genes have been found to be associated to altered sperm parameters, mainly oligozoospermia, indicating a potential involvement of DNA methylation in male infertility. All, but *MTHFR*, gene investigations have been carried out at a single-study level and need further studies to be confirmed. On the contrary, improper *MTHFR* promoter methylation, such as hypermethylation, has been repeatedly found in aberrant sperm samples, suggesting the methylation-induced *MTHFR* dysregulation as a potential causal factor of abnormal sperm changes. Interestingly, *MTHFR* hypermethylation has been found in normal sperm samples of males from idiopathic infertile couples. This may indicate that *MTHFR* dysfunction could play a role in changing sperm function and health, possibly by lowering CpG methylation content in imprinted genes, which, in turn, may affect embryo development and pregnancy outcome (Rotondo et al., 2013). In view of finding gene methylation hallmarks in male infertility, these data are interesting and will deserve further investigations. For instance, large studies of different infertile populations along with a functional analysis of hypermethylation are needed in order to elucidate the role and the mechanisms of the *MTHFR* gene in human spermatogenesis and the related pathologies.

#### Gene Methylation Changes in *LINE*s

The methylation status of *LINE1* in sperm DNA from infertile males was initially evaluated by Kobayashi et al. (2007) who reported no correlation between *LINE1* methylation and male reproductive function. No correlation has also been found in sperm DNA from idiopathic infertile males belonging to recurrent spontaneous miscarriage couples (Ankolkar et al., 2013). Low methylation levels at *LINE1* have been shown in asthenospermic and OAT patients in an additional study, although no statistical significance was found between cases and controls (Boissonnas et al., 2010; Tian et al., 2014). A meta-analysis in 291 infertile patients and 198 fertile individuals from seven published works found no significant correlation with *LINE1* methylation levels (Santi et al., 2017). Conversely, DNA methylation marks at *LINE1* and the repetitive sequences *ALU YB8*, *NBL2*, and *D4Z4* have been found to be significantly lower in spermatozoa from idiopathic infertile males than in somatic cells (Urdinguio et al., 2015). A significant decrease in global 5mC levels and *LINE1* promoter methylation in spermatozoa of males from couples experiencing recurrent pregnancy loss has also been described (Khambata et al., 2021). Similarly, *LINE1* hypomethylation along with promoter hypermethylation-associated silencing of *PIWIL2* and *Tudor Domain Containing 1* genes, two *PIWI/piRNA* pathway-related genes, has been found in sperm DNA from infertile males affected by spermatogenic disorders (Heyn et al., 2012). Animal model studies have reported similar results with other *PIWI/piRNA* pathway-related genes and satellite DNA regions (Capra et al., 2019; Zhang et al., 2020).

*LINE1* methylation levels evaluated through sperm genome methylation analyses might be a good indicator of genome-wide alterations. Indeed, these repetitive sequences have frequently been considered as surrogate markers for global methylation changes (Pinto-Medel et al., 2017). However, the role of *LINE1* methylation in male infertility is far to be elucidated. Few studies are currently available and often reporting contradictory data, probably due to the lack of homogeneity in experimental design and data processing among studies.

### High-Throughput Techniques for DNA Methylome Investigation

Over the years, with the development of molecular technology/methods (Park et al., 2012; Tognon et al., 2015; Rotondo et al., 2018b, 2020a; Mazzoni et al., 2020; Preti et al., 2020; Oton-Gonzalez et al., 2021), many studies have investigated the relationship between DNA methylation and male infertility on a genome-wide scale by employing high-throughput techniques (Houshdaran et al., 2007; Ferfouri et al., 2013; Schütte et al., 2013; Camprubí et al., 2016; Du et al., 2016; Jenkins et al., 2016a, b; Laqqan et al., 2017a; Lee et al., 2019). The first array-wide analysis was performed by Houshdaran et al. (2007) highlighting improper DNA methylation at *repetitive element satellite 2*, *H-RAS*, *neurotrophin* 3, *paired-box gene 8*, and *Stratifin*, *3′-nucleotidase*, and *metallothionein 1A* genes in semen samples with abnormal sperm concentration, motility, and morphology. DNA methylation variations at over 9K CpGs spread throughout the testicular genome have been detected between obstructive and non-obstructive azoospermic patients (Ferfouri et al., 2013). The human methylation 450k array analysis in semen samples from couples who conceived and couples who did not showed significantly decreased and increased methylation in two and three genomic regions, respectively, in the “failure-to-conceive” group. Interestingly, the two sites with low methylation content were at closely related genes known to be expressed in the sperm, i.e., *HSPA1L* and *HSPA1B* (Jenkins et al., 2016b). A recent methylome and transcriptome study in euploid blastocysts derived from couples with OAT males reported significant alterations in approximately 1.1K CpGs compared to the cryopreserved euploid blastocysts used as controls (Denomme et al., 2018). A pathway analysis elucidated the genes involved in the regulation of cellular metabolic process as universally affected. This epigenetic dysregulation provided an explanation for the reduced reproductive potential in OAT patients despite euploid blastocyst transfers (Denomme et al., 2018).

Additional array-wide studies have been reported to show the following: (i) changes in the methylation of testis/epididymis-specific genes in the testis and epididymis of vasectomized males (Wu et al., 2013), (ii) loss of methylation in inflammation- and immune response-related genes in sperm samples from males belonging to infertile couples (Schütte et al., 2013), and (iii) gain of methylation in spermatogenesis-related genes in sperm samples derived from individuals undergoing assisted reproduction (Schütte et al., 2013).

In normospermic males, the DNA methylome of the sperm cell differs between infertile and fertile, and this difference may be predictive of embryo quality during *in vitro* fertilization (Aston et al., 2015). Nevertheless, differences in sperm epigenome within healthy subjects have been also detected, indicating intra-sample heterogeneity in DNA methylation patterns (Jenkins et al., 2014). Furthermore, additional studies have underlined potential epigenetic heterogeneity among, and within, sperm cells, spermatogonia, and gonocytes (Kuhtz et al., 2014; Laurentino et al., 2015, 2016).

The impairment of both sperm epigenome and gene expression in relation to male reproductive dysfunction was recently explored using high-throughput techniques. Hypermethylation-induced silencing at *SRY-Box Transcription Factor 30* gene has been detected in association with non-obstructive azoospermia (Han et al., 2020). Two array-wide studies have described inverse correlations between DNA methylation and the mRNA expression of genes involved in different pathways, such as the response to hormone stimulus, activation of protein kinase activity, apoptosis, and reproduction, in sperm from both severe oligozoospermic and azoospermic patients (Li et al., 2017; Wu et al., 2020).

As high-throughput approaches are able to detect extensive variations in epigenetic marks, DNA methylation changes could potentially be used as a diagnostic tool to predict the risk of both male infertility and male infertility-related diseases (Aston et al., 2015; Fang et al., 2019; Patel et al., 2020). For instance, an array-based DNA methylation profile conducted on peripheral blood has revealed variations in DNA methylation patterns in infertile males compared to normozoospermic fertile controls, suggesting that epigenome-based blood markers can be used for diagnostic purposes (Sarkar et al., 2019). This has also been suggested by the results of another high-throughput study focused on congenital hypopituitarism (CH), which is a pituitary gland hormone deficiency that leads to metabolic disorders and male reproductive dysfunction. This study identified methylation at CpG sites on two spermatogenesis/testicular development-related genes in whole blood DNA isolated from a cohort of CH patients (Fang et al., 2019). In this context, the clinical application of these highly sensitive techniques cannot be ruled out. As high-throughput approaches can potentially support work on defining the forms of male infertility, these methods will deserve attention in andrological clinical practice (Ferlin and Foresta, 2014).

### DNA Methylating and Demethylating Enzymes

DNA methyltransferases and *TET* are two key regulative gene families involved in DNA methylation and demethylation pathways, respectively, during both embryogenesis and spermatogenesis (Nettersheim et al., 2013; Barišić et al., 2017; Li et al., 2019). Despite their critical role in sperm methylation, the *DNMT* and *TET* genes have been poorly investigated in the context of male infertility (Uysal et al., 2016, 2019; Tang et al., 2017, 2018). Decreased expression of DNMT1, -3A, and -3B enzymes in association with global DNA methylation changes has been detected in the testes of non-obstructive azoospermic patients, suggesting that the methylation-induced *DNMT* inactivation may be involved in male reproductive dysfunction (Uysal et al., 2019). The presence of *DNMT1* SNP variant rs4804490 was found to be associated with an increased risk of idiopathic infertility in males with abnormal semen parameters (Tang et al., 2017). In the same study, no associations were afterward determined between additional four DNMT variants, i.e., *DNMT1* (rs4804490), *DNMT3A* (rs1550117), *DNMT3B* (rs2424909), and *DNMT3L* (rs7354779), and aberrant DNA methylation at several imprinted genes, including *H19*, *GNAS complex locus* (*GNAS*), and *GTP-binding protein Di-Ras3* (*DIRAS3*) (Tang et al., 2018). Conversely, two SNP variants, rs2656927/rs8103849, of *ubiquitin-like, containing PHD and RING finger domains 1* (*UHRF1*) gene, which is tightly related to the *DNMT1* pathway, have been identified in the blood of oligozoospermic infertile males (Zhu et al., 2019). Other recent evidence indicated that *UHRF1* inactivation may have a negative impact on male fertility potential, as the conditional loss of this gene in germ cells leads to DNA hypomethylation, upregulation of retrotransposons, and DNA damage response activation (Dong et al., 2019). *UHRF1* also cooperates with the *PIWI/piRNA* pathway during spermatogenesis, suggesting a molecular link between DNA methylation and the *PIWI/piRNA* pathway in the germline (Dong et al., 2019).

Ten-eleven translocation *1*, *-2*, and *-3* genes have been found to be consecutively expressed at different stages of spermatogenesis, and their expression levels have been positively correlated to male reproductive potential and pregnancy (Ni et al., 2016). Indeed sperms from oligozoospermic and/or asthenozoospermia males have significantly reduced TET1, -2 and -3 mRNAs compared to fertile donors, but not to normozoospermic males (Ni et al., 2016). TET1 protein deficiency in spermatogonia decreases the 5hmC levels and downregulates genes that are involved in several pathways, such as cell cycle, germ cell differentiation, meiosis, and reproduction, resulting in the reduction of spermatogonia stem cells and germ cell differentiation (Huang et al., 2020).

When taken together, these studies suggest that *DNMT* and *TET* impairment in sperm cells may represent a potential risk factor for male infertility. Therefore, further investigations are needed to assess the role of DNMT and TET enzymes in the male infertility phenotype.

### Gene Methylation Changes in Imprinted Genes

Imbalances in the methylation patterns of imprinted genes could potentially impair spermatogenesis and/or favor the male infertility phenotype (Hartmann et al., 2006; Rotondo et al., 2012, 2013; Das et al., 2017; Laurentino et al., 2019; Åsenius et al., 2020a; Pohl et al., 2021). The methylation status of a number of imprinted genes has been investigated in relation to impaired spermatogenesis and/or reproductive dysfunction (Table 2). DNA methylation defects of three maternally imprinted genes, i.e., *PLAGL1*, *MEST*, and *DIRAS3*, have been identified in sperm DNAs from infertile males with a low semen concentration (Houshdaran et al., 2007). One of these genes, *MEST*, has been repeatedly found as hypermethylated in association with the male infertility phenotype (Marques et al., 2004; Hammoud et al., 2010; El Hajj et al., 2011; Montjean et al., 2013; Xu et al., 2016). Specifically, the improper DNA methylation at *MEST* has been (i) linked to low sperm concentration and motility as well as poor sperm morphology in idiopathic infertile males (Marques et al., 2008; Poplinski et al., 2010; Kläver et al., 2013), (ii) detected in primary spermatocytes from azoospermic patients presenting complete or incomplete maturation arrest (Marques et al., 2017), (iii) associated with decreased bi-testicular volume and increased follicle-stimulating hormone levels (Kläver et al., 2013), and (iv) associated with abnormal protamine ratio in oligozoospermic patients (Hammoud et al., 2010). These data have been further confirmed by a meta-analytic study (Santi et al., 2017). Recently, *MEST* hypermethylation has been found in the spermatozoa of male partners from couples experiencing recurrent pregnancy loss (Khambata et al., 2021).

**TABLE 2:** Aberrant methylation of imprinted genes associated with abnormalities of semen parameters.

**Gene**	**Imprinted allele**	**Function**	**Associated sperm parameters**	**References**
*CTNNA3*	Paternal	Alpha catenin	Oligo-/oligoasthenozoospermia	Sujit et al. (2018)
*DIRAS3*	Maternal	GTPase	Oligozoospermia	Houshdaran et al. (2007)
*DLGAP2*	Maternal	Membrane-associated protein	Not specified	Schrott et al. (2020)
			Oligo-/oligoasthenozoospermia	Sujit et al. (2018)
GATA3	Maternal	Transcriptional activator	Oligo-/oligoasthenozoospermia	Sujit et al. (2018)
*GNAS*	Maternal/Paternal	G-protein alpha subunit	Oligozoospermia	Tang et al. (2018)
			Oligoasthenozoospermia	Zhang et al. (2019)
*GTL2*	Paternal	Transcription regulator (lncRNA)	Oligozoospermia	Kobayashi et al. (2007)
*H19*	Paternal	Tumor suppressor (lncRNA)	Oligozoospermia	Tang et al. (2018)
			Oligozoospermia	Marques et al. (2008)
			Oligozoospermia	Li et al. (2016)
			Oligoasthenozoospermia	Dong et al. (2017)
			Oligoastheno-/astenoteratozoospermia	Peng et al. (2018)
			Oligoasthenoteratozoospermia	Boissonnas et al. (2010)
			Oligozoospermia	Marques et al. (2004)
*IGF2*	Maternal	Growth factor	Not specified	Poplinski et al. (2010)
			Not specified	Ni et al. (2019)
*IGF2-H19*	Maternal/paternal		Oligo-/asteno-/terato-/oligoteratozoospermia	Rotondo et al. (2013)
			Oligozoospermia	Bruno et al. (2018)
			Asthenozoospermia	Lou et al. (2019)
*KCNQ1*	Maternal	Potassium channel	Not specified	Ni et al. (2019)
*LIT1*	Maternal	Transcription regulator (lncRNA)	Oligozoospermia	Hammoud et al. (2010)
			Oligozoospermia	Kobayashi et al. (2007)
*MAGI2*	Paternal	Membrane-associated protein	Oligo-/oligoasthenozoospermia	Sujit et al. (2018)
*MEG3*	Paternal	Transcription regulator (lncRNA)	Oligozoospermia	Bruno et al. (2018)
*MEST*	Maternal	Hydrolase	Oligozoospermia	Houshdaran et al. (2007)
			Oligozoospermia	Kläver et al. (2013)
			Oligozoospermia	Marques et al. (2008)
			Oligozoospermia	Marques et al. (2004)
			Oligozoospermia	Kobayashi et al. (2007)
			Oligozoospermia	Hammoud et al. (2010)
			Oligoasthenozoospermia	Zhang et al. (2019)
			Not specified	Poplinski et al. (2010)
			Not specified	Khambata et al. (2021)
*PEG3*	Maternal	Zinc finger	Oligozoospermia	Kobayashi et al. (2007)
			Not specified	Khambata et al. (2021)
*PEG10*	Maternal	Zinc finger	Not specified	Khambata et al. (2021)
*SNRPN*	Maternal	Small nuclear ribonucleoprotein	Oligozoospermia	Hammoud et al. (2010)
			Oligoasthenozoospermia	Botezatu et al. (2014)
			Oligoastheno-/astenoteratozoospermia	Peng et al. (2018)
			Astheno-/teratozoospermia	Dong et al. (2017)
*SNURF*	Maternal	SNRPN upstream reading frame	Oligozoospermia	Bruno et al. (2018)
*TP73*	Paternal	Tumor protein	Oligo-/oligoasthenozoospermia	Sujit et al. (2018)
*ZAC*	Maternal	Zinc finger	Oligozoospermia	Kobayashi et al. (2007)
			Not specified	Khambata et al. (2021)
*ZCCHC13*	Maternal	Zinc finger	Non-obstructive azoospermia	Li et al. (2018)

Altered methylation in *H19* has been broadly documented to be associated with infertile males affected by oligozoospermia, asthenozoospermia, teratozoospermia, OA, AT, and OAT (Marques et al., 2004, 2008, 2010; Kobayashi et al., 2007; Boissonnas et al., 2010; Minor et al., 2011; Rotondo et al., 2013; Li et al., 2016; Dong et al., 2017; Bruno et al., 2018; Peng et al., 2018). Idiopathic infertile males with normal semen parameters have also been found to carry methylation defects at *H19* in their sperm (Poplinski et al., 2010; Ankolkar et al., 2013; Tang et al., 2018). Imprinting errors at *H19* gene were detected in primary spermatocytes and elongated spermatids/spermatozoa with incomplete maturation arrest from two azoospermic patients, although the differences in *H19* methylation levels between patients and controls were not statistically significant (Marques et al., 2017). In addition, abnormal methylation at *H19* has been shown at the regulatory region CTCF-binding site 6 (CTCF6), located within the DMR of *IGF2-H19*, in sperm DNA from infertile males (Rotondo et al., 2013). Therefore, sperm cells carrying methylation defects at the *IGF2-H19* CTCF6 region are at a high risk of causing biallelic inactivation of the *IGF2* gene, which, in turn, could negatively impact embryo development and/or pregnancy outcome (Boissonnas et al., 2010; Santi et al., 2017; Bruno et al., 2018). Similarly, methylation defects at *IGF2-H19* have been detected in placenta tissues from offspring conceived by assisted reproductive technologies (ART) (Pinborg et al., 2016; Hattori et al., 2019; Lou et al., 2019). Thus, these studies have pointed out that ART procedures may account for the epigenetic defects in the embryo.

Altered DNA methylation patterns at *overlapping transcript 1* (*LIT1*) and *SNRPN* have been found in infertile males tested with abnormal protamine ratio in sperm cells (Hammoud et al., 2010). Aberrant methylation at the *SNRPN* gene has also been associated with altered sperm motility and morphology as well as with OA and AT (Botezatu et al., 2014; Peng et al., 2018). Interestingly, imprinting errors at the *SNRPN* gene and *IGF2-H19* have also been found in spermatozoa from asthenozoospermia patients and in ART-conceived human fetuses (Lou et al., 2019). Hypermethylation at *SNRPN*, alongside other maternally imprinted genes involved in embryonic germ cell development, such as *MEG3*, *PEG1* (also known as *MEST*), *PEG3*, *LIT1*, and *PLAG1 like zinc finger 1* (*ZAC)*, as well as loss of methylation at the paternal imprinted gene *GTL2* has been detected in sperm DNA derived from patients affected by moderate/severe oligozoospermia (Kobayashi et al., 2007). Hypermethylation of *PEG1*, *PEG3*, *PEG10*, and *ZAC* genes has been shown in spermatozoa from male partners from couples experiencing recurrent pregnancy loss (Khambata et al., 2021). Contrariwise, the lack of altered methylation has been reported for *PEG1*, *ZAC*, and *GTL2* in the sperm of male partners from recurrent spontaneous miscarriages (Ankolkar et al., 2013). Lastly, the maternally imprinted gene *SNRPN upstream reading frame protein*, which is related to the *SNRPN* pathway, has been detected as hypermethylated in association with oligozoospermia (Bruno et al., 2018).

An additional imprinted gene possibly involved in male reproductive dysfunction is *zinc-finger CCHC-type containing 13* (*ZCCHC13*). *ZCCHC13* is a novel imprinted gene involved in an epigenetic mechanism known as X-chromosome inactivation and has been detected both hypermethylated and down-regulated in testis biopsies isolated from non-obstructive azoospermia patients (Li et al., 2018). Low methylation at *GNAS* and *DIRAS3* imprinted genes has also been found to be more prevalent in idiopathic infertile males, especially in oligozoospermic males, than in fertile males (Bruno et al., 2018; Tang et al., 2018). The evidence that improper DNA methylation at the *GNAS* gene is linked to male infertility is also supported by animal models (Congras et al., 2014).

Imprinting errors could also be related to DNA fragmentation in sperm cells. Indeed, the improper methylation of the paternally imprinted gene *Potassium Voltage-Gated Channel Subfamily Q Member 1* (*KCNQ1*), along with that of *IGF2*, has been found to be associated with a high level of DNA fragmentation in semen samples (Ni et al., 2019). In addition, the centromeric (domain 2) differentially methylated region (KvDMR) located in exon 10 of *KCNQ1OT1* has been found to be hypermethylated in the spermatozoa of male partners from couples experiencing recurrent pregnancy loss (Khambata et al., 2021).

A recent array-wide methylation analysis conducted on sperm DNA from a cohort of males identified a number of genes associated with infertility, including the maternally imprinted genes *DLG associated protein 2* (*DLGAP2*) and *GATA binding protein 3* as well as the paternally imprinted genes *catenin alpha 3*, *membrane-associated guanylate kinase*, and *tumor protein 73* (Sujit et al., 2018). An additional gene, *brevican*, which is a non-imprinted gene involved in brain extracellular matrix formation, has been identified in this study. All these genes might be considered as novel potential candidates accounting for male infertility phenotype, and their roles in spermatogenesis need to be further investigated (Sujit et al., 2018).

In conclusion, many imprinted genes have been investigated for methylation in aberrant semen samples, such as oligozoospermia, asthenozoospermia, and teratozoospermia. H19 and MEST were the most investigated genes in aberrant sperm samples, showing repetitively aberrant loss or gain of methylation, respectively, which, in turn, may be causal factors in the development of male infertility. Of note is the fact that aberrant methylations have also been found in infertile males with normal sperm parameters. This aspect may have important implications in the diagnosis of male infertility, which, to date, lacks explanation in 30% of infertile males. It is also hoped that further studies will shed light into the etiology of these aberrant imprinting patterns in paternally or maternally imprinted genes in idiopathic infertility cases. Indeed the loss of methylation of paternally imprinted genes may be due to a deficiency of DNMTs, whereas hypermethylation of maternally imprinted genes might result from erroneous *de novo* methylation or a failure to erase maternal imprint in the male germ cell genomes (Barlow and Bartolomei, 2014; Uysal et al., 2019).

### Lifestyle Factors

Habits and lifestyle factors, such as smoking, alcohol consumption, and diet, have been investigated in an effort to gain insight into the causes of aberrant sperm DNA methylation in relation to male infertility (El Khoury et al., 2019). A recent pilot study conducted on sperm DNA from a cohort of infertile males who underwent a supervised yoga practice regimen reported DNA methylation changes at nearly 400 genes, suggesting a link between positive lifestyle practices and male reproductive health (Bisht et al., 2020).

Tobacco smoke has a strong negative impact on sperm DNA methylation in a number of genes, also demonstrated in animal models (Xu et al., 2013; Dai et al., 2016; Dong et al., 2017; Laqqan et al., 2017a; Hamad et al., 2018; Wyck et al., 2018; Fragou et al., 2019). The imprinted gene *SNRPN* has been found to be aberrantly methylated in asthenozoospermia and teratozoospermic smokers (Dong et al., 2017). Other studies on humans and/or animal models have reported modifications to sperm DNA methylation following cannabis use (Murphy et al., 2018; Reece and Hulse, 2019). Improper *DLGAP2* methylation has recently been reported in sperm from cannabis users, thereby suggesting the potentially negative effect of cannabis use prior to conception on imprinting marks (Schrott et al., 2020). In this context, the impact of parental drug addiction on sperm DNA methylation in drug-sired offspring needs to be more deeply explored (Jarred et al., 2018; Nieto and Kosten, 2019). Moreover, both alcohol and nicotine consumption seem to impair DNA methylation in several genes and genomic regions, leading to male infertility. Indeed data on DNA methylation changes at *cyclin-dependent kinase inhibitor 2A* and *LINE1* and an increased risk of male reproductive dysfunction have been reported (Zhang et al., 2019). More recently, evidence has also indicated that DNA methylation defects at *MEST* and *GNAS* genes, along with altered sperm motility, morphology, and concentration, could be linked to alcohol and nicotine exposure (Zhang et al., 2019).

Paternal diet is able to alter sperm epigenome and has been associated with negative pregnancy outcomes in mice (Lambrot et al., 2013). In addition, sperm from mice under a folate-deficient diet showed differential DNA methylation marks at genes implicated in development, diabetes, autism, and schizophrenia (Lambrot et al., 2013). In humans, several studies have reported on the relationship between paternal and/or maternal diet and sperm DNA methylation in male infertility (Shukla et al., 2014; Hoek et al., 2020; Toschi et al., 2020). A recent study conducted on infertile males who underwent oral supplementation with micronutrients in support of folate, B vitamins, zinc, and cysteines reported an increase in DNA methylation levels, resulting in improved sperm nuclear maturation and antioxidant defenses, with a possible positive effect on reproductive function (Bassiri et al., 2020). Furthermore, evidences on sperm DNA from infertile males indicate a high methylation of *vitamin D receptor* promoter in vitamin D-deficient conditions, thus suggesting the role of diet in epigenetic modifications and male reproductive dysfunction (Vladoiu et al., 2017). Similarly, one investigation indicated that parental diet can modulate the methylation levels of several genes, including liver-specific genes involved in cholesterol/lipid metabolism, whereas no effect of paternal diet on sperm DNA methylation was found in other studies (Carone et al., 2010; Rando and Simmons, 2015; Shea et al., 2015; Whitelaw, 2015). Similarly, parental pre-conceptional obesity could potentially impact on the establishment of imprinting marks during embryogenesis (Soubry et al., 2015; Ou et al., 2019; Åsenius et al., 2020b; Keyhan et al., 2021). Changes in methylation in two imprinted genes, *PLAGL1* and *MEG3*, have been detected in umbilical cord blood leukocytes in a group of newborns from obese mothers, making a link between maternal overnutrition and the impairment of imprinting marks in the offspring (Soubry et al., 2015). In males, imprinted genes involved in growth and development, including *PEG3* and *neuronatin* alongside *MEST*, have been found to be hypomethylated in DNA derived from the blood of children of obese fathers. The relationship between parental obesity and the methylation status of the offspring clearly indicates that spermatogenesis may be susceptible to environmental factors from an epigenetic point of view (Soubry et al., 2015). A recent animal model study supported and extended this conclusion by reporting data about environmental toxicant exposure and transgenerational inheritance of epigenetic marks (Sadler-Riggleman et al., 2019). Additional animal models have shown similar results, reporting that epigenetic alterations at *PEG3* and *H19* can occur in sperm cells in offspring from diabetic and/or obese mice (Ge et al., 2014). These studies, when taken together, highlight that both defective DNA methylation and imprinting marks may be transferred through generations, potentially affecting the health of adult offspring (Grossniklaus et al., 2013; Ge et al., 2014; Soubry et al., 2015; Blanco Rodríguez and Camprubí Sánchez, 2019). However, this assumption has been questioned in a recent meta-analysis of coordinated epigenome-wide association studies of paternal prenatal body mass index (BMI) in relation to DNA methylation (Sharp et al., 2021). The study conclusions do not support the hypothesis that paternal BMI around the time of pregnancy is linked with offspring-blood DNA methylation, even at the imprinted regions (Sharp et al., 2021). Considering the data available, more research is needed to address the role of paternal diet on sperm DNA methylation in infertile males.

## Future Perspectives

This review provides an overview of gene and genome methylation, its regulation during the spermatogenesis process, and the current knowledge on those DNA methylation defects which are known to be involved in male reproductive dysfunctions.

Most of the aforementioned studies have added sufficient strength to the hypothesis that sperm methylation is associated with sperm alterations and infertility. In spite of these reports, the causative role of improper DNA methylation marks in inducing male infertility remains poorly characterized, particularly due to the lack of studies on the mechanisms involving DNA methylation in sperm cells.

It is convincible that aberrations in methylation at specific target genes may reflect whole methylome defects due to altered DNMT and TET activities during sperm cell development and spermatogenesis. On the other hand, several risk factors, such as lifestyle, drugs, hormones, and diet, may account for the gain or loss of methylation in key genes in sperm cells. New functional studies on this topic are needed to better elucidate the mechanisms affecting methylation in sperm. Nevertheless, extensive knowledge of sperm DNA methylation status in association with reduced reproductive capability is useful in developing novel diagnostic tools for male infertility (Balasubramanian et al., 2019; Sarkar et al., 2019). The current primary diagnostic protocol for identifying infertile males relies on the assessment of sperm number, motility, and morphology. This diagnostic approach has several limitations in differentiating fertile and infertile males, as abnormalities causing male reproductive dysfunction are still unknown in a fraction of males. In addition, this approach also provides limited information on male reproductive potential and cannot be employed as a prognostic tool in predicting pregnancy success and possible outcome (Kläver and Gromoll, 2014). When taken together, these drawbacks in the current protocols make DNA methylation evaluation a putative useful tool in clinical practice (Sarkar et al., 2019). A large number of studies have already demonstrated that aberrant DNA methylation in spermatozoa is linked to defective sperm parameters and male infertility phenotype as well as a negative pregnancy outcome. In this context, sperm DNA methylation could become a novel diagnostic and prognostic parameter for assessing male infertility and pregnancy outcome, respectively (Kläver and Gromoll, 2014). This approach has also been reported in monitoring other diseases (Luján et al., 2019). Thanks to high-throughput technology, which is rapidly increasing in epigenetic studies, methylome analyses may soon allow the methylation differences between infertile and fertile males to be characterized, thereby greatly improving the current knowledge on the relationship between sperm DNA methylation and male infertility (Ferlin and Foresta, 2014). Hopefully, the translation of this technology into clinical andrological practice will improve the diagnostic and prognostic assessment of infertile males (Kläver and Gromoll, 2014).

## Conclusion

The role of DNA methylation in spermatogenesis, sperm function, and male infertility is an important research area that deserves attention. Although recent evidence seems to indicate a possible overestimation of aberrant DNA methylation in male infertility (Camprubí et al., 2012; Leitão et al., 2020; Åsenius et al., 2020b), the aforementioned studies have provided significant associations between improper DNA methylation in sperm and infertile males.

A number of genes have been found to be differentially methylated in relation to impaired spermatogenesis and/or reproductive dysfunction. DNA methylation defects in genes, including *MEST* and *H19* within imprinted genes and *MTHFR* within non-imprinted genes, have been previously identified in a wide number of studies, and their defective marks could be potentially employed as useful tools in clinical practice for assessing male infertility (Marques et al., 2004, 2008, 2010; Kobayashi et al., 2007; Boissonnas et al., 2010; Hammoud et al., 2010; El Hajj et al., 2011; Minor et al., 2011; Rotondo et al., 2012, 2013; Montjean et al., 2013; Haggarty, 2015; Dai et al., 2016; Xu et al., 2016; Dong et al., 2017; Li et al., 2017; Bruno et al., 2018; Saraswathy et al., 2018; Coppedè et al., 2019; Mishra et al., 2019; Sarkar et al., 2019).

The lack of studies for several of the additional genes reported herein and/or the conflicting results for others make the use of defective DNA methylation marks as a diagnostic tool still unlikely. Further studies are necessary to identify novel genes that could potentially be employed as tools in clinical practice.

As suggested in several works, the dysregulation of epigenetic mechanisms, including the aberrant methylation of DNA, may play an important role in the development of infertility with unknown etiology in a high fraction of males. However, further research is needed to epigenetically explore the etiology of this disease. Indeed the use of DNA methylation as a marker or cause of sperm abnormalities in the field of fertility is only beginning to be explored thoroughly and is currently not completely clarified. Moreover, the use of methylation changes in DNA as a marker to identify the male infertility factor is difficult, as these changes may have little or even no significant biological impact or multiple different changes may be necessary to establish infertile phenotypes. In this effort, methylation signatures in normal sperm from fertile individuals should provide useful knowledge for elucidating these topics. Moreover, as aging is correlated with changes in DNA methylation, investigating sperm DNA methylation in age-stratified normal fertile individuals should also be taken into account as well as to improve our knowledge in this field (Johnson et al., 2012). Another point deserving attention in the context of methodological approaches is the potential contamination by somatic cells during epigenetic analyses, such as blood, white, and epithelial cells, as a result of incorrect sperm cell isolation/processing (Jenkins et al., 2017). Standardized and detailed protocols for processing sperm samples should be used in studying DNA methylation in male infertility, as even only a few contaminated somatic cells might alter the epigenetic signatures of sperm cells. Research into sperm DNA methylation in male infertility might be an important future area of study. We thus encourage further studies focused on the role of DNA methylation in spermatogenesis and male reproductive potential as well as embryo development and pregnancy outcome. Such newly acquired data could improve the diagnostic and prognostic setup of male infertility phenotypes and pregnancy outcomes, respectively, with high health benefits for humans.

## Author Contributions

JR, MT, and FM contributed to conceptualization and writing—review and editing. JR contributed to writing—original draft preparation. CM and CL contributed to visualization. MT and FM contributed to supervision, contributed to funding acquisition, and project administration. All the authors have read and agreed to the published version of the manuscript.

## Conflict of Interest

The authors declare that the research was conducted in the absence of any commercial or financial relationships that could be construed as a potential conflict of interest.

## Abbreviations

ARTs: assisted reproductive technologies
AT: asthenoteratozoospermia
CH: congenital hypopituitarism
CGIs: CpG islands
CTCF6: CTCF-binding site 6
DMRs: differentially methylated regions
DNMTs: DNA methyltransferases
ICEs: imprint control elements
ICRs: imprinting control regions
LINEs: long interspersed nuclear elements
OA: oligoasthenozoospermia
OT: oligoteratozoospermia
OAT: oligoasthenoteratozoospermia
PGCs: primordial germ cells
TET: ten-eleven translocation.

## References

[B1] Abrao M. S. Muzii L. Marana R. (2013). Anatomical causes of female infertility and their management. *Int. J. Gynaecol. Obstet.* 123 Suppl 2 S18–S24. 10.1016/j.ijgo.2013.09.008 24119894

[B2] Agarwal A. Mulgund A. Hamada A. Chyatte M. R. (2015). A unique view on male infertility around the globe. *Reprod. Biol. Endocrinol.* 13:37. 10.1186/s12958-015-0032-1 25928197 PMC4424520

[B3] Alkhaled Y. Laqqan M. Tierling S. Lo Porto C. Hammadeh M. E. (2018). DNA methylation level of spermatozoa from subfertile and proven fertile and its relation to standard sperm parameters. *Andrologia* 50:e13011. 10.1111/and.13011 29574923

[B4] Altakroni B. Nevin C. Carroll M. Murgatroyd C. Horne G. Brison D. R. (2021). The marker of alkyl DNA base damage, N7-methylguanine, is associated with semen quality in men. *Sci. Rep.* 11:3121. 10.1038/s41598-021-81674-x 33542261 PMC7862252

[B5] Ankolkar M. Salvi V. Warke H. Vundinti B. R. Balasinor N. H. (2013). Methylation status of imprinted genes DLK1-GTL2, MEST (PEG1), ZAC (PLAGL1), and LINE-1 elements in spermatozoa of normozoospermic men, unlike H19 imprinting control regions, is not associated with idiopathic recurrent spontaneous miscarriages. *Fertil. Steril.* 99 1668–1673. 10.1016/j.fertnstert.2013.01.107 23415968

[B6] Aravin A. A. Sachidanandam R. Bourc’his D. Schaefer C. Pezic D. Toth K. F. (2008). A piRNA pathway primed by individual transposons is linked to de novo DNA methylation in mice. *Mol. Cell* 31 785–799. 10.1016/j.molcel.2008.09.003 18922463 PMC2730041

[B7] Arnaud P. (2010). Genomic imprinting in germ cells: imprints are under control. *Reproduction* 140 411–423. 10.1530/REP-10-0173 20501788

[B8] Åsenius F. Danson A. F. Marzi S. J. (2020a). DNA methylation in human sperm: a systematic review. *Hum. Reprod. Update* 26 841–873. 10.1093/humupd/dmaa025 32790874

[B9] Åsenius F. Gorrie-Stone T. J. Brew A. Panchbhaya Y. Williamson E. Schalkwyk L. C. (2020b). The DNA methylome of human sperm is distinct from blood with little evidence for tissue-consistent obesity associations. *PLoS Genet.* 16:e1009035. 10.1371/journal.pgen.1009035 33048947 PMC7584170

[B10] Asim A. Agarwal S. Panigrahi I. Saiyed N. Bakshi S. (2017). MTHFR promoter hypermethylation may lead to congenital heart defects in down syndrome. *Intractable Rare Dis. Res.* 6 295–298. 10.5582/irdr.2017.01068 29259859 PMC5735284

[B11] Aston K. I. Punj V. Liu L. Carrell D. T. (2012). Genome-wide sperm deoxyribonucleic acid methylation is altered in some men with abnormal chromatin packaging or poor in vitro fertilization embryogenesis. *Fertil. Steril.* 97 285–292. 10.1016/j.fertnstert.2011.11.008 22154369

[B12] Aston K. I. Uren P. J. Jenkins T. G. Horsager A. Cairns B. R. Smith A. D. (2015). Aberrant sperm DNA methylation predicts male fertility status and embryo quality. *Fertil. Steril.* 104 1388–1397. 10.1016/j.fertnstert.2015.08.019 26361204

[B13] Balasubramanian A. Thirumavalavan N. Pastuszak A. W. (2019). DNA methylation profiling of peripheral blood samples is a promising new approach to screen for male infertility. *Fertil. Steril.* 112 32–33. 10.1016/j.fertnstert.2019.04.019 31133383 PMC7333522

[B14] Barau J. Teissandier A. Zamudio N. Roy S. Nalesso V. Hérault Y. (2016). The DNA methyltransferase DNMT3C protects male germ cells from transposon activity. *Science* 354 909–912. 10.1126/science.aah5143 27856912

[B15] Barišić A. Pereza N. Hodžić A. Ostojić S. Peterlin B. (2017). A single nucleotide polymorphism of DNA methyltransferase 3B gene is a risk factor for recurrent spontaneous abortion. *Am. J. Reprod. Immunol.* 78:e12765. 10.1111/aji.12765 28940947

[B16] Barlow D. P. Bartolomei M. S. (2014). Genomic imprinting in mammals. *Cold Spring Harb. Perspect. Biol.* 6:a018382. 10.1101/cshperspect.a018382 24492710 PMC3941233

[B17] Bassiri F. Tavalaee M. Dattilio M. Nasr-Esfahani M. H. (2020). Micronutrients in support to the carbon cycle activate antioxidant defences and reduce sperm DNA damage in infertile men attending assisted reproductive technology programs: clinical trial study. *Int. J. Fertil. Steril.* 14 57–62. 10.22074/ijfs.2020.6084 32112637 PMC7139231

[B18] Baylin S. B. Jones P. A. (2011). A decade of exploring the cancer epigenome-biological and translational implications. *Nat. Rev. Cancer* 11 726–734. 10.1038/nrc3130 21941284 PMC3307543

[B19] Benchaib M. Braun V. Ressnikof D. Lornage J. Durand P. Niveleau A. (2005). Influence of global sperm DNA methylation on IVF results. *Hum. Reprod.* 30 768–773. 10.1093/humrep/deh684 15640258

[B20] Biermann K. Steger K. (2007). Epigenetics in male germ cells. *J. Androl.* 28 466–480. 10.2164/jandrol.106.002048 17287457

[B21] Bisht S. Banu S. Srivastava S. Pathak R. U. Kumar R. Dada R. (2020). Sperm methylome alterations following yoga-based lifestyle intervention in patients of primary male infertility: a pilot study. *Andrologia* 2:e13551. 10.1111/and.13551 32124461

[B22] Blanco Rodríguez J. Camprubí Sánchez C. (2019). Epigenetic transgenerational inheritance. *Adv. Exp. Med. Biol.* 1166 57–74. 10.1007/978-3-030-21664-1_4 31301046

[B23] Boissonnas C. C. El Abdalaoui H. Haelewyn V. Fauque P. Dupont J. M. Gut I. (2010). Specific epigenetic alterations of IGF2-H19 locus in spermatozoa from infertile men. *Eur. J. Hum. Genet.* 18 73–80. 10.1038/ejhg.2009.117 19584898 PMC2987171

[B24] Botezatu A. Socolov R. Socolov D. Iancu I. V. Anton G. (2014). Methylation pattern of methylene tetrahydrofolate reductase and small nuclear ribonucleoprotein polypeptide N promoters in oligoasthenospermia: a case-control study. *Reprod. Biomed. Online* 28 225–231. 10.1016/j.rbmo.2013.10.010 24365028

[B25] Broad K. D. Curley J. P. Keverne E. B. (2009). Increased apoptosis during neonatal brain development underlies the adult behavioral deficits seen in mice lacking a functional paternally expressed gene 3 (Peg3). *Dev. Neurobiol.* 69 314–325. 10.1002/dneu.20702 19224563

[B26] Bruno C. Blagoskonov O. Barberet J. Guilleman M. Daniel S. Tournier B. (2018). Sperm imprinting integrity in seminoma patients? *Clin. Epigenetics* 10:125. 10.1186/s13148-018-0559-z 30340650 PMC6194738

[B27] Camprubí C. Pladevall M. Grossmann M. Garrido N. Pons M. C. Blanco J. (2012). Semen samples showing an increased rate of spermatozoa with imprinting errors have a negligible effect in the outcome of assisted reproduction techniques. *Epigenetics* 7 1115–1124. 10.4161/epi.21743 22885410 PMC3469453

[B28] Camprubí C. Salas-Huetos A. Aiese-Cigliano R. Godo A. Pons M. C. Castellano G. (2016). Spermatozoa from infertile patients exhibit differences of DNA methylation associated with spermatogenesis-related processes: an array-based analysis. *Reprod. Biomed. Online* 33 709–719. 10.1016/j.rbmo.2016.09.001 27692602

[B29] Cannarella R. Condorelli R. A. La Vignera S. Bellucci C. Luca G. Calafiore R. (2019). IGF2 and IGF1R mRNAs are detectable in human spermatozoa. *World J. Mens Health* 1 37–47. 10.5534/wjmh.190070 31496145 PMC7502314

[B30] Capra E. Lazzari B. Turri F. Cremonesi P. Portela A. M. R. Ajmone-Marsan P. (2019). Epigenetic analysis of high and low motile sperm populations reveals methylation variation in satellite regions within the pericentromeric position and in genes functionally related to sperm DNA organization and maintenance in *Bos taurus*. *BMC Genomics* 20:940. 10.1186/s12864-019-6317-6 31810461 PMC6898967

[B31] Carone B. R. Fauquier L. Habib N. Shea J. M. Hart C. E. Li R. (2010). Paternally induced transgenerational environmental reprogramming of metabolic gene expression in mammals. *Cell* 143 1084–1096. 10.1016/j.cell.2010.12.008 21183072 PMC3039484

[B32] Carrell D. T. (2019). The sperm epigenome: implications for assisted reproductive technologies. *Adv. Exp. Med. Biol.* 1166 47–57. 10.1007/978-3-030-21664-1_3 31301045

[B33] Castillo J. Jodar M. Oliva R. (2018). The contribution of human sperm proteins to the development and epigenome of the preimplantation embryo. *Hum. Reprod. Update* 24 535–555. 10.1093/humupd/dmy017 29800303

[B34] Chen Z. (2001). Mice deficient in methylenetetrahydrofolate reductase exhibit hyperhomocysteinemia and decreased methylation capacity, with neuropathology and aortic lipid deposition. *Hum. Mol. Genet.* 10 433–443. 10.1093/hmg/10.5.433 11181567

[B35] Cho C. Jung-Ha H. Willis W. D. Goulding E. H. Stein P. Xu Z. (2003). Protamine 2 deficiency leads to sperm DNA damage and embryo death in Mice1. *Biol. Reprod.* 69 211–217. 10.1095/biolreprod.102.015115 12620939

[B36] Choi Y. Kim J. O. Shim S. H. Lee Y. Kim J. H. Jeon Y. J. (2016). Genetic variation of methylenetetrahydrofolate reductase (MTHFR) and thymidylate synthase (TS) genes is associated with idiopathic recurrent implantation failure. *PLoS One* 11:e0160884. 10.1371/journal.pone.0160884 27560137 PMC4999086

[B37] Congras A. Yerle-Bouissou M. Pinton A. Vignoles F. Liaubet L. Ferchaud S. (2014). Sperm DNA methylation analysis in swine reveals conserved and species-specific methylation patterns and highlights an altered methylation at the GNAS locus in infertile boars1. *Biol. Reprod.* 91:137. 10.1095/biolreprod.114.119610 25320151

[B38] Contini C. Rotondo J. C. Magagnoli F. Maritati M. Seraceni S. Graziano A. (2018). Investigation on silent bacterial infections in specimens from pregnant women affected by spontaneous miscarriage. *J. Cell. Physiol.* 234 100–107. 10.1002/jcp.26952 30078192

[B39] Cooper T. G. Noonan E. von Eckardstein S. Auger J. Baker H. W. G. Behre H. M. (2009). World Health Organization reference values for human semen characteristics. *Hum. Reprod. Update* 16 231–246. 10.1093/humupd/dmp048 19934213

[B40] Coppedè F. Denaro M. Tannorella P. Migliore L. (2016). Increased MTHFR promoter methylation in mothers of down syndrome individuals. *Mutat. Res.* 787 1–6. 10.1016/j.mrfmmm.2016.02.008 26926955

[B41] Coppedè F. Stoccoro A. Tannorella P. Gallo R. Nicolì V. Migliore L. (2019). Association of polymorphisms in genes involved in one-carbon metabolism with MTHFR methylation levels. *Int. J. Mol. Sci.* 20:3754. 10.3390/ijms20153754 31370354 PMC6696388

[B42] Craig J. R. Jenkins T. G. Carrell D. T. Hotaling J. M. (2017). Obesity, male infertility, and the sperm epigenome. *Fertil. Steril.* 107 848–859. 10.1016/j.fertnstert.2017.02.115 28366411

[B43] Dai J. Xu W. Zhao X. Zhang M. Zhang D. Nie D. (2016). Protein profile screening: reduced expression of sord in the mouse epididymis induced by nicotine inhibits tyrosine phosphorylation level in capacitated spermatozoa. *Reproduction* 151 227–237. 10.1530/REP-15-0370 26647419

[B44] Das L. Parbin S. Pradhan N. Kausar C. Patra S. K. (2017). Epigenetics of reproductive infertility. *Front. Biosci. (Schol. Ed.)* 9:509–535. 10.2741/s497 28410129

[B45] Denomme M. M. McCallie B. R. Parks J. C. Booher K. Schoolcraft W. B. Katz-Jaffe M. G. (2018). Inheritance of epigenetic dysregulation from male factor infertility has a direct impact on reproductive potential. *Fertil. Steril.* 110 419–428. 10.1016/j.fertnstert.2018.04.004 29961538

[B46] Dong H. Wang Y. Zou Z. Chen L. Shen C. Xu S. (2017). Abnormal methylation of imprinted genes and cigarette smoking: assessment of their association with the risk of male infertility. *Reprod. Sci.* 24 114–123. 10.1177/1933719116650755 27247128

[B47] Dong J. Wang X. Cao C. Wen Y. Sakashita A. Chen S. (2019). UHRF1 suppresses retrotransposons and cooperates with PRMT5 and PIWI proteins in male germ cells. *Nat. Commun.* 17:4705. 10.1038/s41467-019-12455-4 31624244 PMC6797737

[B48] Du Y. Li M. Chen J. Duan Y. Wang X. Qiu Y. (2016). Promoter targeted bisulfite sequencing reveals DNA methylation profiles associated with low sperm motility in asthenozoospermia. *Hum. Reprod.* 31 24–33. 10.1093/humrep/dev283 26628640

[B49] El Hajj N. Zechner U. Schneider E. Tresch A. Gromoll J. Hahn T. (2011). Methylation status of imprinted genes and repetitive elements in sperm DNA from infertile males. *Sex. Dev.* 5 60–69. 10.1159/000323806 21293114

[B50] El Khoury D. Fayjaloun S. Nassar M. Sahakian J. Aad P. Y. (2019). Updates on the effect of mycotoxins on male reproductive efficiency in mammals. *Toxins* 11:515. 10.3390/toxins11090515 31484408 PMC6784030

[B51] Fang X. Chen C. Cai J. Xiang E. Li J. Chen P. (2019). Genome-wide methylation study of whole blood cells DNA in men with congenital hypopituitarism disease. *Int. J. Mol. Med.* 43 155–166. 10.3892/ijmm.2018.3945 30365064 PMC6257856

[B52] Fend-Guella D. L. Von Kopylow K. Spiess A. N. Schulze W. Salzbrunn A. Diederich S. (2019). The DNA methylation profile of human spermatogonia at single-cell- and single-allele-resolution refutes its role in spermatogonial stem cell function and germ cell differentiation. *Mol. Hum. Reprod.* 25 283–294. 10.1093/molehr/gaz017 30892608

[B53] Ferfouri F. Boitrelle F. Ghout I. Albert M. Molina Gomes D. Wainer R. (2013). A genome-wide DNA methylation study in azoospermia. *Andrology* 1 815–821. 10.1111/j.2047-2927.2013.00117.x 23996935

[B54] Ferlin A. Foresta C. (2014). New genetic markers for male infertility. *Curr. Opin. Obstet. Gynecol.* 3 193–198. 10.1097/GCO.0000000000000061 24743183

[B55] Födinger M. Hörl W. H. Sunder-Plassmann G. (2000). Molecular biology of 5,10-methylenetetrahydrofolate reductase. *J. Nephrol.* 13 20–33. 10720211

[B56] Fragou D. Pakkidi E. Aschner M. Samanidou V. Kovatsi L. (2019). Smoking and DNA methylation: correlation of methylation with smoking behavior and association with diseases and fetus development following prenatal exposure. *Food Chem. Toxicol.* 129 312–327. 10.1016/j.fct.2019.04.059 31063835

[B57] Franzago M. La Rovere M. Franchi P. G. Vitacolonna E. Stuppia L. (2019). Epigenetics and human reproduction: the primary prevention of the noncommunicable diseases. *Epigenomics* 11 1441–1460. 10.2217/epi-2019-0163 31596147

[B58] Gao Z. Moorjani P. Sasani T. A. Pedersen B. S. Quinlan A. R. Jorde L. B. (2019). Overlooked roles of DNA damage and maternal age in generating human germline mutations. *Proc. Natl. Acad. Sci. U. S. A.* 116 9491–9500. 10.1073/pnas.1901259116 31019089 PMC6511033

[B59] Ge Z. J. Liang Q. X. Hou Y. Han Z. M. Schatten H. Sun Q. Y. (2014). Maternal obesity and diabetes may cause DNA methylation alteration in the spermatozoa of offspring in mice. *Reprod. Biol. Endocrinol.* 12:29. 10.1186/1477-7827-12-29 24721882 PMC3984639

[B60] Giacone F. Cannarella R. Mongioì L. M. Alamo A. Condorelli R. A. Calogero A. E. (2019). Epigenetics of male fertility: effects on assisted reproductive techniques. *World J. Mens Health* 37 148–156. 10.5534/wjmh.180071 30588778 PMC6479088

[B61] Gkountela S. Zhang K. X. Shafiq T. A. Liao W. W. Hargan-Calvopiña J. Chen P. Y. (2015). DNA demethylation dynamics in the human prenatal germline. *Cell* 161 1425–1436. 10.1016/j.cell.2015.05.012 26004067 PMC4458157

[B62] Greenberg M. V. C. Bourc’his D. (2019). The diverse roles of DNA methylation in mammalian development and disease. *Nat. Rev. Mol. Cell Biol.* 20 590–607. 10.1038/s41580-019-0159-6 31399642

[B63] Grossniklaus U. Kelly B. Ferguson-Smith A. C. Pembrey M. Lindquist S. (2013). Transgenerational epigenetic inheritance: how important is it? *Nat. Rev. Genet.* 14 228–235. 10.1038/nrg3435 23416892 PMC4066847

[B64] Gunes S. Agarwal A. Henkel R. Mahmutoglu A. M. Sharma R. Esteves S. C. (2018a). Association between promoter methylation of MLH1 and MSH2 and reactive oxygen species in oligozoospermic men—a pilot study. *Andrologia* 50:e12903. 10.1111/and.12903 28983945

[B65] Gunes S. Al-Sadaan M. Agarwal A. (2015). Spermatogenesis, DNA damage and DNA repair mechanisms in male infertility. *Reprod. Biomed. Online* 31 309–319. 10.1016/j.rbmo.2015.06.010 26206278

[B66] Gunes S. Arslan M. A. Hekim G. N. T. Asci R. (2016). The role of epigenetics in idiopathic male infertility. *J. Assist. Reprod. Genet.* 33 553–569. 10.1007/s10815-016-0682-8 26941097 PMC4870443

[B67] Gunes S. Kablan A. Agarwal A. Henkel R. (2018b). “Epigenetics, spermatogenesis, and male infertility,” in *Reproductomics: The -Omics Revolution and Its Impact on Human Reproductive Medicine*, eds Horcajadas J. A. Gosálvez J. (Cambridge, MA: Academic Press) 171–187. 10.1016/B978-0-12-812571-7.00011-3

[B68] Guo G. Chmielecki J. Goparaju C. Heguy A. Dolgalev I. Carbone M. (2015). Whole-exome sequencing reveals frequent genetic alterations in BAP1, NF2, CDKN2A, and CUL1 in malignant pleural mesothelioma. *Cancer Res.* 75 264–269. 10.1158/0008-5472.CAN-14-1008 25488749

[B69] Gupta N. Gupta S. Dama M. David A. Khanna G. Khanna A. (2011). Strong association of 677 C>T substitution in the MTHFR gene with male infertility – a study on an Indian population and a meta-analysis. *PLoS One* 6:e22277. 10.1371/journal.pone.0022277 21799811 PMC3140509

[B70] Habib W. A. Brioude F. Azzi S. Rossignol S. Linglart A. Sobrier M. L. (2019). Transcriptional profiling at the DLK1/MEG3 domain explains clinical overlap between imprinting disorders. *Sci. Adv.* 5:9425. 10.1126/sciadv.aau9425 30801013 PMC6382400

[B71] Haggarty P. (2015). Genetic and metabolic determinants of human epigenetic variation. *Curr. Opin. Clin. Nutr. Metab. Care* 18 334–338. 10.1097/MCO.0000000000000194 26049630

[B72] Hamad M. F. Dayyih W. A. A. Laqqan M. AlKhaled Y. Montenarh M. Hammadeh M. E. (2018). The status of global DNA methylation in the spermatozoa of smokers and non-smokers. *Reprod. Biomed. Online* 37 581–589. 10.1016/j.rbmo.2018.08.016 30366840

[B73] Hammoud S. S. Purwar J. Pflueger C. Cairns B. R. Carrell D. T. (2010). Alterations in sperm DNA methylation patterns at imprinted loci in two classes of infertility. *Fertil. Steril.* 94 1728–1733. 10.1016/j.fertnstert.2009.09.010 19880108

[B74] Han F. Jiang X. Li Z. Zhuang X. Zhang X. Ouyang W. (2020). Epigenetic inactivation of SOX30 is associated with male infertility and offers a therapy target for non-obstructive azoospermia. *Mol. Ther. Nucleic Acids* 6 72–83. 10.1016/j.omtn.2019.10.038 31835093 PMC6926170

[B75] Hanna C. W. Demond H. Kelsey G. (2018). Epigenetic regulation in development: is the mouse a good model for the human? *Hum. Reprod. Update* 24 556–576. 10.1093/humupd/dmy021 29992283 PMC6093373

[B76] Hartmann S. Bergmann M. Bohle R. M. Weidner W. Steger K. (2006). Genetic imprinting during impaired spermatogenesis. *Mol. Hum. Reprod.* 12 407–411. 10.1093/molehr/gal040 16608903

[B77] Hattori H. Hiura H. Kitamura A. Miyauchi N. Kobayashi N. Takahashi S. (2019). Association of four imprinting disorders and ART. *Clin. Epigenetics* 11:21. 10.1186/s13148-019-0623-3 30732658 PMC6367766

[B78] Hekim N. Gunes S. Asci R. Henkel R. Abur U. (2021). Semiquantitative promoter methylation of MLH1 and MSH2 genes and their impact on sperm DNA fragmentation and chromatin condensation in infertile men. *Andrologia* 53:e13827. 10.1111/and.13827 33112435

[B79] Heyn H. Ferreira H. J. Bassas L. Bonache S. Sayols S. Sandoval J. (2012). Epigenetic disruption of the PIWI pathway in human spermatogenic disorders. *PLoS One* 6 1–18. 10.1371/journal.pone.0047892 23112866 PMC3480440

[B80] Higashimoto K. Urano T. Sugiura K. Yatsuki H. Joh K. Zhao W. (2003). Loss of CpG methylation is strongly correlated with loss of histone H3 Lysine 9 methylation at DMR-LIT1 in patients with beckwith-wiedemann syndrome. *Am. J. Hum. Genet.* 73 948–956. 10.1086/378595 12949703 PMC1180615

[B81] Hoek J. Steegers-Theunissen R. P. M. Willemsen S. P. Schoenmakers S. (2020). Paternal folate status and sperm quality, pregnancy outcomes, and epigenetics: a systematic review and meta-analysis. *Mol. Nutr. Food Res.* 64:e1900696. 10.1002/mnfr.201900696 32032459 PMC7317557

[B82] Houshdaran S. Cortessis V. K. Siegmund K. Yang A. Laird P. W. Sokol R. Z. (2007). Widespread epigenetic abnormalities suggest a broad DNA methylation erasure defect in abnormal human sperm. *PLoS One* 2:e1289. 10.1371/journal.pone.0001289 18074014 PMC2100168

[B83] Huang G. Liu L. Wang H. Gou M. Gong P. Tian C. (2020). Tet1 deficiency leads to premature reproductive aging by reducing spermatogonia stem cells and germ cell differentiation. *iScience* 23:100908. 10.1016/j.isci.2020.100908 32114381 PMC7049665

[B84] Huang Y. Liu H. Du H. Zhang W. Kang X. Luo Y. (2019). Developmental features of DNA methylation in CpG islands of human gametes and preimplantation embryos. *Exp. Ther. Med.* 4447–4456. 10.3892/etm.2019.7523 31105782 PMC6507515

[B85] Ilacqua A. Izzo G. Pietro Emerenziani G. Baldari C. Aversa A. (2018). Lifestyle and fertility: the influence of stress and quality of life on male fertility. *Reprod. Biol. Endocrinol.* 16:115. 10.1186/s12958-018-0436-9 30474562 PMC6260894

[B86] Jarred E. G. Bildsoe H. Western P. S. (2018). Out of sight, out of mind? Germ cells and the potential impacts of epigenomic drugs [version 1; referees: 3 approved]. *F1000Res.* 7:F1000. 10.12688/f1000research.15935.1 30613387 PMC6305226

[B87] Jenkins T. G. Aston K. I. Hotaling J. M. Shamsi M. B. Simon L. Carrell D. T. (2016a). Teratozoospermia and asthenozoospermia are associated with specific epigenetic signatures. *Andrology* 4 843–849. 10.1111/andr.12231 27529490

[B88] Jenkins T. G. Aston K. I. James E. R. Carrell D. T. (2017). Sperm epigenetics in the study of male fertility, offspring health, and potential clinical applications. *Syst. Biol. Reprod. Med.* 63 69–76. 10.1080/19396368.2016.1274791 28301256

[B89] Jenkins T. G. Aston K. I. Meyer T. D. Hotaling J. M. Shamsi M. B. Johnstone E. B. (2016b). Decreased fecundity and sperm DNA methylation patterns. *Fertil. Steril.* 105 51–57. 10.1016/j.fertnstert.2015.09.013 26453269 PMC4890464

[B90] Jenkins T. G. Aston K. I. Trost C. Farley J. Hotaling J. M. Carrell D. T. (2014). Intra-sample heterogeneity of sperm DNA methylation. *Mol. Hum. Reprod.* 21 313–319. 10.1093/molehr/gau115 25542834

[B91] Jenkins T. G. Carrell D. T. (2011). The paternal epigenome and embryogenesis: poising mechanisms for development. *Asian J. Androl.* 13 76–80. 10.1038/aja.2010.61 20972451 PMC3739388

[B92] Jin C. Qin T. Barton M. C. Jelinek J. Issa J. P. J. (2015). Minimal role of base excision repair in TET-induced global DNA demethylation in HEK293T cells. *Epigenetics* 10 1006–1013. 10.1080/15592294.2015.1091145 26440216 PMC4844212

[B93] Johnson A. A. Akman K. Calimport S. R. G. Wuttke D. Stolzing A. de Magalhães J. P. (2012). The role of DNA methylation in aging, rejuvenation, and age-related disease. *Rejuvenation Res.* 15 483–494. 10.1089/rej.2012.1324 23098078 PMC3482848

[B94] Kabartan E. Gunes S. Arslan M. A. Asci R. (2019). Investigating the relationship between BRCA1 and BRCA2 genes methylation profile and sperm DNA fragmentation in infertile men. *Andrologia* 51:e13308. 10.1111/and.13308 31095775

[B95] Karaca M. Z. Konac E. Yurteri B. Bozdag G. Sogutdelen E. Bilen C. Y. (2017). Association between methylenetetrahydrofolate reductase (MTHFR) gene promoter hypermethylation and the risk of idiopathic male infertility. *Andrologia* 49:e12698. 10.1111/and.12698 27596009

[B96] Keyhan S. Burke E. Schrott R. Huang Z. Grenier C. Price T. (2021). Male obesity impacts DNA methylation reprogramming in sperm. *Clin. Epigenetics* 13:17. 10.1186/s13148-020-00997-0 33494820 PMC7831195

[B97] Khambata K. Raut S. Deshpande S. Mohan S. Sonawane S. Gaonkar R. (2021). DNA methylation defects in spermatozoa of male partners from couples experiencing recurrent pregnancy loss. *Hum. Reprod. Oxf. Engl.* 36 48–60. 10.1093/humrep/deaa278 33319906

[B98] Khazamipour N. Noruzinia M. Fatehmanesh P. Keyhanee M. Pujol P. (2009). MTHFR promoter hypermethylation in testicular biopsies of patients with non-obstructive azoospermia: the role of epigenetics in male infertility. *Hum. Reprod.* 24 2361–2364. 10.1093/humrep/dep194 19477879

[B99] Kitamura A. Miyauchi N. Hamada H. Hiura H. Chiba H. Okae H. (2015). Epigenetic alterations in sperm associated with male infertility. *Congenit. Anom.* 55 133–144. 10.1111/cga.12113 26212350

[B100] Kläver R. Gromoll J. (2014). Bringing epigenetics into the diagnostics of the andrology laboratory: challenges and perspectives. *Asian J. Androl.* 16 669–674. 10.4103/1008-682X.125412 24923457 PMC4215682

[B101] Kläver R. Tüttelmann F. Bleiziffer A. Haaf T. Kliesch S. Gromoll J. (2013). DNA methylation in spermatozoa as a prospective marker in andrology. *Andrology* 1 731–740. 10.1111/j.2047-2927.2013.00118.x 23970452

[B102] Kobayashi H. Sato A. Otsu E. Hiura H. Tomatsu C. Utsunomiya T. (2007). Aberrant DNA methylation of imprinted loci in sperm from oligospermic patients. *Hum. Mol. Genet.* 16 2542–2551. 10.1093/hmg/ddm187 17636251

[B103] Kovac J. R. Khanna A. Lipshultz L. I. (2015). The effects of cigarette smoking on male fertility. *Postgrad. Med.* 127 338–341. 10.1080/00325481.2015.1015928 25697426 PMC4639396

[B104] Krausz C. (2011). Male infertility: pathogenesis and clinical diagnosis. *Best Pract. Res. Clin. Endocrinol. Metab.* 127 271–285. 10.1016/j.beem.2010.08.006 21397198

[B105] Kuhtz J. Schneider E. El Hajj N. Zimmermann L. Fust O. Linek B. (2014). Epigenetic heterogeneity of developmentally important genes in human sperm: implications for assisted reproduction outcome. *Epigenetics* 9 1648–1658. 10.4161/15592294.2014.988063 25625849 PMC4622742

[B106] Kulac T. Hekim N. Kocamanoglu F. Beyaz C. Gunes S. Asci R. (2021). Methylation patterns of methylenetetrahydrofolate reductase gene promoter in infertile males. *Andrologia* 53:e13942. 10.1111/and.13942 33372270

[B107] Kuramochi-Miyagawa S. Watanabe T. Gotoh K. Totoki Y. Toyoda A. Ikawa M. (2008). DNA methylation of retrotransposon genes is regulated by Piwi family members MILI and MIWI2 in murine fetal testes. *Genes Dev.* 22 908–917. 10.1101/gad.1640708 18381894 PMC2279202

[B108] Lambrot R. Xu C. Saint-Phar S. Chountalos G. Cohen T. Paquet M. (2013). Low paternal dietary folate alters the mouse sperm epigenome and is associated with negative pregnancy outcomes. *Nat. Commun.* 4:2889. 10.1038/ncomms3889 24326934 PMC3863903

[B109] Lanzillotti C. De Mattei M. Mazziotta C. Taraballi F. Rotondo J. C. Tognon M. (2021). Long non-coding RNAs and microRNAs interplay in osteogenic differentiation of mesenchymal stem cells. *Front. Cell Dev. Biol.* 9:646032. 10.3389/fcell.2021.646032 33898434 PMC8063120

[B110] Laqqan M. Tierling S. Alkhaled Y. Lo Porto C. Solomayer E. F. Hammadeh M. (2017a). Spermatozoa from males with reduced fecundity exhibit differential DNA methylation patterns. *Andrology* 5 971–978. 10.1111/andr.12362 28544631

[B111] Laqqan M. Tierling S. Alkhaled Y. Lo Porto C. Solomayer E. F. Hammadeh M. E. (2017b). Aberrant DNA methylation patterns of human spermatozoa in current smoker males. *Reprod. Toxicol.* 71 126–133. 10.1016/j.reprotox.2017.05.010 28576685

[B112] Laurentino S. Beygo J. Nordhoff V. Kliesch S. Wistuba J. Borgmann J. (2015). Epigenetic germline mosaicism in infertile men. *Hum. Mol. Genet.* 24 1295–1304. 10.1093/hmg/ddu540 25336341

[B113] Laurentino S. Borgmann J. Gromoll J. (2016). On the origin of sperm epigenetic heterogeneity. *Reproduction* 151 71–78. 10.1530/REP-15-0436 26884419

[B114] Laurentino S. Heckmann L. Di Persio S. Li X. Zu Hörste G. M. Wistuba J. (2019). High-resolution analysis of germ cells from men with sex chromosomal aneuploidies reveals normal transcriptome but impaired imprinting. *Clin. Epigenetics* 11:127. 10.1186/s13148-019-0720-3 PMC6714305 31462300

[B115] Lee S. W. Hwang H. H. Hsu P. W. C. Chuang T. Y. Liu C. W. Wu L. S. H. (2019). Whole-genome methylation profiling from PBMCs in acute-exacerbation COPD patients with good and poor responses to corticosteroid treatment. *Genomics* 111 1381–1386. 10.1016/j.ygeno.2018.09.010 30248490

[B116] Leitão E. Di Persio S. Laurentino S. Wöste M. Dugas M. Kliesch S. (2020). The sperm epigenome does not display recurrent epimutations in patients with severely impaired spermatogenesis. *Clin. Epigenetics* 12:61. 10.1186/s13148-020-00854-0 32375885 PMC7204326

[B117] Lévesque N. Leclerc D. Gayden T. Lazaris A. De Jay N. Petrillo S. (2016). Murine diet/tissue and human brain tumorigenesis alter Mthfr/MTHFR 5′-end methylation. *Mamm. Genome* 27 122–134. 10.1007/s00335-016-9624-0 26951114

[B118] Li E. (2002). Chromatin modification and epigenetic reprogramming in mammalian development. *Nat. Rev. Genet.* 3 662–673. 10.1038/nrg887 12209141

[B119] Li X. P. Hao C. L. Wang Q. Yi X. M. Jiang Z. S. (2016). H19 gene methylation status is associated with male infertility. *Exp. Ther. Med.* 12 451–456. 10.3892/etm.2016.3314 27347077 PMC4907102

[B120] Li Z. Chen S. Yang Y. Zhuang X. Tzeng C. M. (2018). Novel biomarker ZCCHC13 revealed by integrating DNA methylation and mRNA expression data in non-obstructive azoospermia. *Cell Death Discov.* 4 10.1038/s41420-018-0033-x 29531833 PMC5841273

[B121] Li Z. Fang F. Zhao Q. Li H. Xiong C. (2019). Supplementation of vitamin C promotes early germ cell specification from human embryonic stem cells. *Stem Cell Res. Ther.* 10:324. 10.1186/s13287-019-1427-2 31730021 PMC6858754

[B122] Li Z. Zhuang X. Zeng J. Tzeng C. M. (2017). Integrated analysis of DNA methylation and mRNA expression profiles to identify key genes in severe oligozoospermia. *Front. Physiol.* 8:261. 10.3389/fphys.2017.00261 28553232 PMC5427114

[B123] Lobo J. Nunes S. P. Gillis A. J. M. Barros-Silva D. Miranda-Gonçalves V. van den Berg A. (2019). XIST-promoter demethylation as tissue biomarker for testicular germ cell tumors and spermatogenesis quality. *Cancers* 11:1385. 10.3390/cancers11091385 31533343 PMC6769809

[B124] Lopomo A. Ricciardi R. Maestri M. Rosa A. Melfi F. Lucchi M. (2016). Gene-specific methylation analysis in thymomas of patients with myasthenia gravis. *Int. J. Mol. Sci.* 17:2121. 10.3390/ijms17122121 27999265 PMC5187921

[B125] Lou H. Le F. Hu M. Yang X. Li L. Wang L. (2019). Aberrant DNA methylation of IGF2-H19 locus in human fetus and in spermatozoa from assisted reproductive technologies. *Reprod. Sci.* 26 997–1004. 10.1177/1933719118802052 30270743

[B126] Louie K. Minor A. Ng R. Poon K. Chow V. Ma S. (2016). Evaluation of DNA methylation at imprinted DMRs in the spermatozoa of oligozoospermic men in association with MTHFR C677T genotype. *Andrology* 4 825–831. 10.1111/andr.12240 27369467

[B127] Luján S. Caroppo E. Niederberger C. Arce J. C. Sadler-Riggleman I. Beck D. (2019). Sperm DNA methylation epimutation biomarkers for male infertility and FSH therapeutic responsiveness. *Sci. Rep.* 14:16786. 10.1038/s41598-019-52903-1 31727924 PMC6856367

[B128] Marcho C. Oluwayiose O. A. Pilsner J. R. (2019). The preconception environment and sperm epigenetics. *Andrology* 8 924–942. 10.1111/andr.12753 31901222 PMC7346722

[B129] Marques C. J. Carvalho F. Sousa M. Barros A. (2004). Genomic imprinting in disruptive spermatogenesis. *Lancet* 363 1700–1702. 10.1016/S0140-6736(04)16256-9 15158633

[B130] Marques C. J. Costa P. Vaz B. Carvalho F. Fernandes S. Barros A. (2008). Abnormal methylation of imprinted genes in human sperm is associated with oligozoospermia. *Mol. Hum. Reprod.* 14 67–74. 10.1093/molehr/gam093 18178607

[B131] Marques C. J. Francisco T. Sousa S. Carvalho F. Barros A. Sousa M. (2010). Methylation defects of imprinted genes in human testicular spermatozoa. *Fertil. Steril.* 94 585–594. 10.1016/j.fertnstert.2009.02.051 19338988

[B132] Marques P. I. Fernandes S. Carvalho F. Barros A. Sousa M. Marques C. J. (2017). DNA methylation imprinting errors in spermatogenic cells from maturation arrest azoospermic patients. *Andrology* 5 451–459. 10.1111/andr.12329 28296202

[B133] Mayer W. Hemberger M. Frank H. G. Grümmer R. Winterhager E. Kaufmann P. (2000). Expression of the imprinted genes MEST/Mest in human and murine placenta suggests a role in angiogenesis. *Dev. Dyn.* 217 1–10. 10.1002/(SICI)1097-0177(200001)217:1<1::AID-DVDY1<3.0.CO;2-4 10679925

[B134] Mazzoni E. Pellegrinelli E. Mazziotta C. Lanzillotti C. Rotondo J. C. Bononi I. (2020). Mother-to-child transmission of oncogenic polyomaviruses BKPyV, JCPyV and SV40. *J. Infect.* 80 563–570. 10.1016/j.jinf.2020.02.006 32097686

[B135] McSwiggin H. M. O’Doherty A. M. (2018). Epigenetic reprogramming during spermatogenesis and male factor infertility. *Reproduction* 156 9–21. 10.1530/REP-18-0009 29717022

[B136] Melamed P. Yosefzon Y. David C. Tsukerman A. Pnueli L. (2018). Tet enzymes, variants, and differential effects on function. *Front. Cell Dev. Biol.* 6:22. 10.3389/fcell.2018.00022 29556496 PMC5844914

[B137] Metin Mahmutoglu A. Gunes S. Asci R. Henkel R. Aydin O. (2021). Association of XRCC1 and ERCC2 promoters’ methylation with chromatin condensation and sperm DNA fragmentation in idiopathic oligoasthenoteratozoospermic men. *Andrologia* 53:e13925. 10.1111/and.13925 33355950

[B138] Miller D. Brinkworth M. Iles D. (2009). Paternal DNA packaging in spermatozoa: more than the sum of its parts? *Reprod. Camb. Engl.* 139 287–301. 10.1530/REP-09-0281 19759174

[B139] Minor A. Chow V. Ma S. (2011). Aberrant DNA methylation at imprinted genes in testicular sperm retrieved from men with obstructive azoospermia and undergoing vasectomy reversal. *Reproduction* 141 749–757. 10.1530/REP-11-0008 21389080

[B140] Mishra J. Talwar S. Kaur L. Chandiok K. Yadav S. Puri M. (2019). Differential global and MTHFR gene specific methylation patterns in preeclampsia and recurrent miscarriages: a case-control study from North India. *Gene* 704 68–73. 10.1016/j.gene.2019.04.036 30986448

[B141] Monk M. Boubelik M. Lehnert S. (1987). Temporal and regional changes in DNA methylation in the embryonic, extraembryonic and germ cell lineages during mouse embryo development. *Development* 99 371–382. 3653008 10.1242/dev.99.3.371

[B142] Montjean D. Benkhalifa M. Dessolle L. Cohen-Bacrie P. Belloc S. Siffroi J. P. (2011). Polymorphisms in MTHFR and MTRR genes associated with blood plasma homocysteine concentration and sperm counts. *Fertil. Steril.* 95 635–640. 10.1016/j.fertnstert.2010.08.054 20888556

[B143] Montjean D. Ravel C. Benkhalifa M. Cohen-Bacrie P. Berthaut I. Bashamboo A. (2013). Methylation changes in mature sperm deoxyribonucleic acid from oligozoospermic men: assessment of genetic variants and assisted reproductive technology outcome. *Fertil. Steril.* 100 1241–1247. 10.1016/j.fertnstert.2013.06.047 23916795

[B144] Moore L. D. Le T. Fan G. (2013). DNA methylation and its basic function. *Neuropsychopharmacology* 38 23–38. 10.1038/npp.2012.112 22781841 PMC3521964

[B145] Murphy L. E. Mills J. L. Molloy A. M. Qian C. Carter T. C. Strevens H. (2011). Folate and vitamin B12 in idiopathic male infertility. *Asian J. Androl.* 13 856–861. 10.1038/aja.2011.96 21857689 PMC3372894

[B146] Murphy S. K. Itchon-Ramos N. Visco Z. Huang Z. Grenier C. Schrott R. (2018). Cannabinoid exposure and altered DNA methylation in rat and human sperm. *Epigenetics* 13 1559–1230. 10.1080/15592294.2018.1554521 30521419 PMC6986792

[B147] Nagamori I. Kobayashi H. Nishimura T. Yamagishi R. Katahira J. Kuramochi-Miyagawa S. (2018). Relationship between PIWIL4-mediated H3K4me2 demethylation and piRNA-dependent DNA methylation. *Cell Rep.* 25 350–356. 10.1016/j.celrep.2018.09.017 30304676

[B148] Nanassy L. Carrell D. T. (2011). Abnormal methylation of the promoter of CREM is broadly associated with male factor infertility and poor sperm quality but is improved in sperm selected by density gradient centrifugation. *Fertil. Steril.* 95 2310–2314. 10.1016/j.fertnstert.2011.03.096 21507395

[B149] Nasri F. Gharesi-Fard B. Namavar Jahromi B. Farazi-fard M. A. Banaei M. Davari M. (2017). Sperm DNA methylation of H19 imprinted gene and male infertility. *Andrologia* 49:e12766. 10.1111/and.12766 28295500

[B150] Navarro-Costa P. Nogueira P. Carvalho M. Leal F. Cordeiro I. Calhaz-Jorge C. (2010). Incorrect DNA methylation of the DAZL promoter CpG island associates with defective human sperm. *Hum. Reprod. Oxf. Engl.* 25 2647–2654. 10.1093/humrep/deq200 20685756 PMC2939755

[B151] Nettersheim D. Heukamp L. C. Fronhoffs F. Grewe M. J. Haas N. Waha A. (2013). Analysis of TET expression/activity and 5mC oxidation during normal and malignant germ cell development. *PLoS One* 8:e82881. 10.1371/journal.pone.0082881 24386123 PMC3873252

[B152] Ni K. Dansranjavin T. Rogenhofer N. Oeztuerk N. Deuker J. Bergmann M. (2016). TET enzymes are successively expressed during human spermatogenesis and their expression level is pivotal for male fertility. *Hum. Reprod.* 31 1411–1424. 10.1093/humrep/dew096 27141042

[B153] Ni W. Pan C. Pan Q. Fei Q. Huang X. Zhang C. (2019). Methylation levels of IGF2 and KCNQ1 in spermatozoa from infertile men are associated with sperm DNA damage. *Andrologia* 51:e13239. 10.1111/and.13239 30680773

[B154] Nieto S. J. Kosten T. A. (2019). Who’s your daddy? Behavioral and epigenetic consequences of paternal drug exposure. *Int. J. Dev. Neurosci.* 78 109–121. 10.1016/j.ijdevneu.2019.07.002 31301337 PMC7448378

[B155] Oton-Gonzalez L. Rotondo J. C. Cerritelli L. Malagutti N. Lanzillotti C. Bononi I. (2021). Association between oncogenic human papillomavirus type 16 and Killian polyp. *Infect. Agent. Cancer* 16:3. 10.1186/s13027-020-00342-3 33413530 PMC7792173

[B156] Ou X. H. Zhu C. C. Sun S. C. (2019). Effects of obesity and diabetes on the epigenetic modification of mammalian gametes. *J. Cell. Physiol.* 234 7847–7855. 10.1002/jcp.27847 30536398

[B157] Park E. Gong E.-Y. Romanelli M. G. Lee K. (2012). Suppression of estrogen receptor-alpha transactivation by thyroid transcription factor-2 in breast cancer cells. *Biochem. Biophys. Res. Commun.* 421 532–537. 10.1016/j.bbrc.2012.04.039 22521644

[B158] Parry L. Clarke A. R. (2011). The roles of the methyl-CpG binding proteins in cancer. *Genes Cancer* 2 618–630. 10.1177/1947601911418499 21941618 PMC3174265

[B159] Patel D. P. Jenkins T. G. Aston K. I. Guo J. Pastuszak A. W. Hanson H. A. (2020). Harnessing the full potential of reproductive genetics and epigenetics for male infertility in the era of “big data.” *Fertil. Steril.* 113 478–488. 10.1016/j.fertnstert.2020.01.001 32089255 PMC7333523

[B160] Peng H. Zhao P. Liu J. Zhang J. Zhang J. Wang Y. (2018). Novel epigenomic biomarkers of male infertility identified by methylation patterns of CpG sites within imprinting control regions of H19 and SNRPN genes. *OMICS J. Integr. Biol.* 22 354–364. 10.1089/omi.2018.0019 29708855

[B161] Peters J. (2000). Imprinting: silently crossing the boundary. *Genome Biol.* 1:REVIEWS1028. 10.1186/gb-2000-1-5-reviews1028 PMC138886 11178262

[B162] Pinborg A. Loft A. Romundstad L. B. Wennerholm U. B. Söderström-Anttila V. Bergh C. (2016). Epigenetics and assisted reproductive technologies. *Acta Obstet. Gynecol. Scand.* 15 12–25. 10.1111/aogs.12799 26458360

[B163] Pinto-Medel M. J. Oliver-Martos B. Urbaneja-Romero P. Hurtado-Guerrero I. Ortega-Pinazo J. Serrano-Castro P. (2017). Global methylation correlates with clinical status in multiple sclerosis patients in the first year of IFNbeta treatment. *Sci. Rep.* 7:8727. 10.1038/s41598-017-09301-2 28821874 PMC5562733

[B164] Pisarska M. D. Chan J. L. Lawrenson K. Gonzalez T. L. Wang E. T. (2019). Genetics and epigenetics of infertility and treatments on outcomes. *J. Clin. Endocrinol. Metab.* 104 1871–1886. 10.1210/jc.2018-01869 30561694 PMC6463256

[B165] Pohl E. Gromoll J. Wistuba J. Laurentino S. (2021). Healthy ageing and spermatogenesis. *Reprod. Camb. Engl.* 161 R89–R101. 10.1530/REP-20-0633 33574214

[B166] Poorang S. Abdollahi S. Anvar Z. Tabei S. M. B. Jahromi B. N. Moein-Vaziri N. (2018). The impact of methylenetetrahydrofolate reductase (MTHFR) sperm methylation and variants on semen parameters and the chance of recurrent pregnancy loss in the couple. *Clin. Lab.* 67 1121–1128. 10.7754/Clin.Lab.2018.171231 30146842

[B167] Poplinski A. Tüttelmann F. Kanber D. Horsthemke B. Gromoll J. (2010). Idiopathic male infertility is strongly associated with aberrant methylation of MEST and IGF2/H19 ICR1. *Int. J. Androl.* 33 642–649. 10.1111/j.1365-2605.2009.01000.x 19878521

[B168] Preti M. Rotondo J. C. Holzinger D. Micheletti L. Gallio N. McKay-Chopin S. (2020). Role of human papillomavirus infection in the etiology of vulvar cancer in Italian women. *Infect. Agent. Cancer* 15:20. 10.1186/s13027-020-00286-8 32266002 PMC7110671

[B169] Rando O. J. Simmons R. A. (2015). I’m eating for two: parental dietary effects on offspring metabolism. *Cell* 161 93–105. 10.1016/j.cell.2015.02.021 25815988 PMC4465102

[B170] Reece A. S. Hulse G. K. (2019). Impacts of cannabinoid epigenetics on human development: reflections on Murphy et al. ‘cannabinoid exposure and altered DNA methylation in rat and human sperm’ epigenetics 2018; 13: 1208-1221. *Epigenetics* 14 1041–1056. 10.1080/15592294.2019.1633868 31293213 PMC6773386

[B171] Reik W. Walter J. (2001). Genomic imprinting: parental influence on the genome. *Nat. Rev. Genet.* 2 21–32. 10.1038/35047554 11253064

[B172] Richardson M. E. Bleiziffer A. Tüttelmann F. Gromoll J. Wilkinson M. F. (2014). Epigenetic regulation of the RHOX homeobox gene cluster and its association with human male infertility. *Hum. Mol. Genet.* 23 12–23. 10.1093/hmg/ddt392 23943794 PMC3857941

[B173] Rose N. R. Klose R. J. (2014). Understanding the relationship between DNA methylation and histone lysine methylation. *Biochim. Biophys. Acta Gene Regul. Mech.* 1839 1362–1372. 10.1016/j.bbagrm.2014.02.007 24560929 PMC4316174

[B174] Rotondo J. C. Aquila G. Oton-Gonzalez L. Selvatici R. Rizzo P. De Mattei M. (2021). Methylation of SERPINA1 gene promoter may predict chronic obstructive pulmonary disease in patients affected by acute coronary syndrome. *Clin. Epigenetics* 13:79. 10.1186/s13148-021-01066-w 33858475 PMC8048251

[B175] Rotondo J. C. Borghi A. Selvatici R. Magri E. Bianchini E. Montinari E. (2016). Hypermethylation-induced inactivation of the IRF6 gene as a possible early event in progression of vulvar squamous cell carcinoma associated with lichen sclerosus. *JAMA Dermatol.* 152 928–933. 10.1001/jamadermatol.2016.1336 27223861

[B176] Rotondo J. C. Borghi A. Selvatici R. Mazzoni E. Bononi I. Corazza M. (2018a). Association of retinoic acid receptor β gene with onset and progression of lichen sclerosus-associated vulvar squamous cell carcinoma. *JAMA Dermatol.* 154 819–823. 10.1001/jamadermatol.2018.1373 29898214 PMC6128494

[B177] Rotondo J. C. Bosi S. Bazzan E. Di Domenico M. De Mattei M. Selvatici R. (2012). Methylenetetrahydrofolate reductase gene promoter hypermethylation in semen samples of infertile couples correlates with recurrent spontaneous abortion. *Hum. Reprod.* 27 3632–3638. 10.1093/humrep/des319 23010533

[B178] Rotondo J. C. Giari L. Guerranti C. Tognon M. Castaldelli G. Fano E. A. (2018b). Environmental doses of perfluorooctanoic acid change the expression of genes in target tissues of common carp. *Environ. Toxicol. Chem.* 37 942–948. 10.1002/etc.4029 29105837

[B179] Rotondo J. C. Oton-Gonzalez L. Mazziotta C. Lanzillotti C. Iaquinta M. R. Tognon M. (2020a). Simultaneous detection and viral DNA load quantification of different Human Papillomavirus types in clinical specimens by the high analytical droplet digital PCR method. *Front. Microbiol.* 11:591452. 10.3389/fmicb.2020.591452 in press 33329471 PMC7710522

[B180] Rotondo J. C. Oton-Gonzalez L. Selvatici R. Rizzo P. Pavasini R. Campo G. C. (2020b). SERPINA1 gene promoter is differentially methylated in peripheral blood mononuclear cells of pregnant women. *Front. Cell Dev. Biol.* 8:550543. 10.3389/fcell.2020.550543 33015055 PMC7494783

[B181] Rotondo J. C. Selvatici R. Di Domenico M. Marci R. Vesce F. Tognon M. (2013). Methylation loss at H19 imprinted gene correlates with methylenetetrahydrofolate reductase gene promoter hypermethylation in semen samples from infertile males. *Epigenetics* 8 990–997. 10.4161/epi.25798 23975186 PMC3883776

[B182] Rousseaux S. Caron C. Govin J. Lestrat C. Faure A. K. Khochbin S. (2005). Establishment of male-specific epigenetic information. *Gene* 345 139–153. 10.1016/j.gene.2004.12.004 15716030

[B183] Sadler-Riggleman I. Klukovich R. Nilsson E. Beck D. Xie Y. Yan W. (2019). Epigenetic transgenerational inheritance of testis pathology and Sertoli cell epimutations: generational origins of male infertility. *Environ. Epigenetics* 29:dvz013. 10.1093/eep/dvz013 31528361 PMC6736068

[B184] Safarinejad M. R. Shafiei N. Safarinejad S. (2011). Relationship between genetic polymorphisms of methylenetetra-hydrofolate reductase (C677T, A1298C, and G1793A) as risk factors for idiopathic male infertility. *Reprod. Sci.* 18 304–315. 10.1177/1933719110385135 20978181

[B185] Santi D. De Vincentis S. Magnani E. Spaggiari G. (2017). Impairment of sperm DNA methylation in male infertility: a meta-analytic study. *Andrology* 5 695–703. 10.1111/andr.12379 28718528

[B186] Santos F. Dean W. (2004). Epigenetic reprogramming during early development in mammals. *Reproduction* 127 643–651. 10.1530/rep.1.00221 15175501

[B187] Saraswathy K. Kaur L. Talwar S. Mishra J. Huidrom S. Sachdeva M. (2018). Methylenetetrahydrofolate reductase gene-specific methylation and recurrent miscarriages: a case-control study from North India. *J. Hum. Reprod. Sci.* 11 142–147. 10.4103/jhrs.JHRS_145_17 30158810 PMC6094536

[B188] Sarkar S. Sujit K. M. Singh V. Pandey R. Trivedi S. Singh K. (2019). Array-based DNA methylation profiling reveals peripheral blood differential methylation in male infertility. *Fertil. Steril.* 112 61–72. 10.1016/j.fertnstert.2019.03.020 31103287

[B189] Schrott R. Acharya K. Itchon-Ramos N. Hawkey A. B. Pippen E. Mitchell J. T. (2020). Cannabis use is associated with potentially heritable widespread changes in autism candidate gene DLGAP2 DNA methylation in sperm. *Epigenetics* 15 161–173. 10.1080/15592294.2019.1656158 31451081 PMC6961656

[B190] Schütte B. El Hajj N. Kuhtz J. Nanda I. Gromoll J. Hahn T. (2013). Broad DNA methylation changes of spermatogenesis, inflammation and immune response-related genes in a subgroup of sperm samples for assisted reproduction. *Andrology* 1 822–829. 10.1111/j.2047-2927.2013.00122.x 23996961 PMC4033565

[B191] Seisenberger S. Andrews S. Krueger F. Arand J. Walter J. Santos F. (2012). The dynamics of genome-wide DNA methylation reprogramming in mouse primordial germ cells. *Mol. Cell* 48 849–862. 10.1016/j.molcel.2012.11.001 23219530 PMC3533687

[B192] Sharma P. Ghanghas P. Kaushal N. Kaur J. Kaur P. (2019). Epigenetics and oxidative stress: a twin-edged sword in spermatogenesis. *Andrologia* 5:e13432. 10.1111/and.13432 31583745

[B193] Sharp G. C. Alfano R. Ghantous A. Urquiza J. Rifas-Shiman S. L. Page C. M. (2021). Paternal body mass index and offspring DNA methylation: findings from the PACE consortium. *Int. J. Epidemiol.* 10.1093/ije/dyaa267 33517419 PMC8407864

[B194] Shea J. M. Serra R. W. Carone B. R. Shulha H. P. Kucukural A. Ziller M. J. (2015). Genetic and epigenetic variation, but not diet, shape the sperm methylome. *Dev. Cell* 35 750–758. 10.1016/j.devcel.2015.11.024 26702833 PMC4691283

[B195] Shukla K. K. Chambial S. Dwivedi S. Misra S. Sharma P. (2014). Recent scenario of obesity and male fertility. *Andrology* 2 809–819. 10.1111/andr.270 25269421

[B196] Solyom S. Kazazian H. H. (2012). Mobile elements in the human genome: implications for disease. *Genome Med.* 4:12. 10.1186/gm311 22364178 PMC3392758

[B197] Soubry A. Murphy S. K. Wang F. Huang Z. Vidal A. C. Fuemmeler B. F. (2015). Newborns of obese parents have altered DNA methylation patterns at imprinted genes. *Int. J. Obes.* 39 650–657. 10.1038/ijo.2013.193 24158121 PMC4048324

[B198] Stangler Herodež Š Zagradišnik B. Erjavec Škerget A. Zagorac A. Takač I. Vlaisavljević V. (2013). MTHFR C677T and A1298C genotypes and haplotypes in slovenian couples with unexplained infertility problems and in embryonic tissues from spontaneous abortions. *Balkan J. Med. Genet.* 16 31–40. 10.2478/bjmg-2013-0015 24265582 PMC3835294

[B199] Stomper J. Rotondo J. C. Greve G. Lübbert M. (2021). Hypomethylating agents (HMA) for the treatment of acute myeloid leukemia and myelodysplastic syndromes: mechanisms of resistance and novel HMA-based therapies. *Leukemia* 10.1038/s41375-021-01218-0 [Epub ahead of print] 33958699 PMC8257497

[B200] Stouffs K. Seneca S. Lissens W. (2014). Genetic causes of male infertility. *Ann. Endocrinol.* 75 109–111. 10.1016/j.ando.2014.03.004 24768008

[B201] Stuppia L. Franzago M. Ballerini P. Gatta V. Antonucci I. (2015). Epigenetics and male reproduction: the consequences of paternal lifestyle on fertility, embryo development, and children lifetime health. *Clin. Epigenetics* 7:120. 10.1186/s13148-015-0155-4 26566402 PMC4642754

[B202] Sujit K. M. Sarkar S. Singh V. Pandey R. Agrawal N. K. Trivedi S. (2018). Genome-wide differential methylation analyses identifies methylation signatures of male infertility. *Hum. Reprod.* 33 2256–2267. 10.1093/humrep/dey319 30358834

[B203] Sujit K. M. Singh V. Trivedi S. Singh K. Gupta G. Rajender S. (2020). Increased DNA methylation in the spermatogenesis-associated (SPATA) genes correlates with infertility. *Andrology* 8 602–609. 10.1111/andr.12742 31838782

[B204] Tang Q. Chen Y. Wu W. Ding H. Xia Y. Chen D. (2017). Idiopathic male infertility and polymorphisms in the DNA methyltransferase genes involved in epigenetic marking. *Sci. Rep.* 7:11219. 10.1038/s41598-017-11636-9 28894282 PMC5593912

[B205] Tang Q. Pan F. Yang J. Fu Z. Lu Y. Wu X. (2018). Idiopathic male infertility is strongly associated with aberrant DNA methylation of imprinted loci in sperm: a case-control study. *Clin. Epigenetics* 10:134. 10.1186/s13148-018-0568-y 30373665 PMC6206675

[B206] Tang W. W. C. Dietmann S. Irie N. Leitch H. G. Floros V. I. Bradshaw C. R. (2015). A unique gene regulatory network resets the human germline epigenome for development. *Cell* 161 1453–1467. 10.1016/j.cell.2015.04.053 26046444 PMC4459712

[B207] Tara S. S. Ghaemimanesh F. Zarei S. Reihani-Sabet F. Pahlevanzadeh Z. Modarresi M. H. (2015). Methylenetetrahydrofolate reductase C677T and A1298C polymorphisms in male partners of recurrent miscarriage couples. *J. Reprod. Infertil.* 16 193–198. 27110516 PMC4819207

[B208] Tian M. Bao H. Martin F. L. Zhang J. Liu L. Huang Q. (2014). Association of DNA methylation and mitochondrial DNA copy number with human semen Quality1. *Biol. Reprod.* 91 1–8. 10.1095/biolreprod.114.122465 25210131

[B209] Tognon M. Luppi M. Corallini A. Taronna A. Barozzi P. Rotondo J. C. (2015). Immunologic evidence of a strong association between non-Hodgkin lymphoma and simian virus 40. *Cancer* 121 2618–2626. 10.1002/cncr.29404 25877010

[B210] Tognon M. Tagliapietra A. Magagnoli F. Mazziotta C. Oton-Gonzalez L. Lanzillotti C. (2020). Investigation on spontaneous abortion and human papillomavirus infection. *Vaccines* 8:473. 10.3390/vaccines8030473 PMC7563606 32854278

[B211] Toschi P. Capra E. Anzalone D. A. Lazzari B. Turri F. Pizzi F. (2020). Maternal peri-conceptional undernourishment perturbs offspring sperm methylome. *Reprod. Camb. Engl.* 159 513–523. 10.1530/REP-19-0549 32103819

[B212] Ullah N. Mansoor A. Micheal S. Mirza B. Qamar R. Mazhar K. (2019). MTHFR polymorphisms as risk for male infertility in Pakistan and its comparison with socioeconomic status in the world. *Pers. Med.* 16 35–49. 10.2217/pme-2018-0045 30468411

[B213] Urdinguio R. G. Bayón G. F. Dmitrijeva M. Toraño E. G. Bravo C. Fraga M. F. (2015). Aberrant DNA methylation patterns of spermatozoa in men with unexplained infertility. *Hum. Reprod.* 30 1014–1028. 10.1093/humrep/dev053 25753583

[B214] Uysal F. Akkoyunlu G. Ozturk S. (2016). DNA methyltransferases exhibit dynamic expression during spermatogenesis. *Reprod. Biomed. Online* 33 690–702. 10.1016/j.rbmo.2016.08.022 27687053

[B215] Uysal F. Akkoyunlu G. Ozturk S. (2019). Decreased expression of DNA methyltransferases in the testes of patients with non-obstructive azoospermia leads to changes in global DNA methylation levels. *Reprod. Fertil. Dev.* 10.1071/rd18246 [Epub ahead of print] 31030726

[B216] Valinluck V. Sowers L. C. (2007). Endogenous cytosine damage products alter the site selectivity of human DNA maintenance methyltransferase DNMT1. *Cancer Res.* 67 946–950. 10.1158/0008-5472.CAN-06-3123 17283125

[B217] Vladoiu S. Botezatu A. Anton G. Manda D. Paun D. L. Oros S. (2017). The involvement of vdr promoter methylation, CDX-2 VDR polymorphism and vitamin d levels in male infertility. *Acta Endocrinol. (Copenh.)* 13 294–301. 10.4183/aeb.2017.294 31149190 PMC6516584

[B218] Von Meyenn F. Reik W. (2015). Forget the parents: epigenetic reprogramming in human germ cells. *Cell* 161 1248–1251. 10.1016/j.cell.2015.05.039 26046435

[B219] Wasserzug-Pash P. Klutstein M. (2019). Epigenetic changes in mammalian gametes throughout their lifetime: the four seasons metaphor. *Chromosoma* 128 423–441. 10.1007/s00412-019-00704-w 31030260

[B220] Whitelaw E. (2015). Sperm DNA methylation: not a vehicle for dietary reprogramming of offspring? *Dev. Cell* 25 668–669. 10.1016/j.devcel.2015.12.005 26702825

[B221] Williams K. Christensen J. Helin K. (2012). DNA methylation: TET proteins-guardians of CpG islands? *EMBO Rep.* 13 28–35. 10.1038/embor.2011.233 22157888 PMC3246258

[B222] Wu C. Ding X. Li H. Zhu C. Xiong C. (2013). Genome-wide promoter methylation profile of human testis and epididymis: identified from cell-free seminal DNA. *BMC Genomics* 14:288. 10.1186/1471-2164-14-288 23622456 PMC3653781

[B223] Wu W. Shen O. Qin Y. Niu X. Lu C. Xia Y. (2010). Idiopathic male infertility is strongly associated with aberrant promoter methylation of methylenetetrahydrofolate reductase (MTHFR). *PLoS One* 5:e13884. 10.1371/journal.pone.0013884 21085488 PMC2976703

[B224] Wu X. Luo C. Hu L. Chen X. Chen Y. Fan J. (2020). Unraveling epigenomic abnormality in azoospermic human males by WGBS, RNA-Seq, and transcriptome profiling analyses. *J. Assist. Reprod. Genet.* 37 789–802. 10.1007/s10815-020-01716-7 32056059 PMC7183037

[B225] Wyck S. Herrera C. Requena C. E. Bittner L. Hajkova P. Bollwein H. (2018). Oxidative stress in sperm affects the epigenetic reprogramming in early embryonic development. *Epigenetics Chromatin* 11:60. 10.1186/s13072-018-0224-y 30333056 PMC6192351

[B226] Xu J. Zhang A. Zhang Z. Wang P. Qian Y. He L. (2016). DNA methylation levels of imprinted and nonimprinted genes DMRs associated with defective human spermatozoa. *Andrologia* 48 1027–1035. 10.1111/and.12535 26804237

[B227] Xu W. Fang P. Zhu Z. Dai J. Nie D. Chen Z. (2013). Cigarette smoking exposure alters Pebp1 DNA methylation and protein profile involved in MAPK signaling pathway in mice Testis1. *Biol. Reprod.* 89 1–11. 10.1095/biolreprod.113.111245 24198121

[B228] Yang F. Wang P. J. (2016). Multiple LINEs of retrotransposon silencing mechanisms in the mammalian germline. *Semin. Cell Dev. Biol.* 59 118–125. 10.1016/j.semcdb.2016.03.001 26957474 PMC5011444

[B229] Yao C. Liu Y. Sun M. Niu M. Yuan Q. Hai Y. (2015). MicroRNAs and DNA methylation as epigenetic regulators of mitosis, meiosis and spermiogenesis. *Reproduction* 150 25–34. 10.1530/REP-14-0643 25852155

[B230] Yin Y. Morgunova E. Jolma A. Kaasinen E. Sahu B. Khund-Sayeed S. (2017). Impact of cytosine methylation on DNA binding specificities of human transcription factors. *Science* 356:eaaj2239. 10.1126/science.aaj2239 28473536 PMC8009048

[B231] Zhang G. W. Wang L. Chen H. Guan J. Wu Y. Zhao J. (2020). Promoter hypermethylation of PIWI/piRNA pathway genes associated with diminished pachytene piRNA production in bovine hybrid male sterility. *Epigenetics* 15 914–931. 10.1080/15592294.2020.1738026 32141383 PMC7518677

[B232] Zhang W. Li M. Sun F. Xu X. Zhang Z. Liu J. (2019). Association of sperm methylation at LINE-1, four candidate genes, and nicotine/alcohol exposure with the risk of infertility. *Front. Genet.* 18:1001. 10.3389/fgene.2019.01001 31681430 PMC6813923

[B233] Zhao L. Zhang S. An X. Tan W. Tang B. Zhang X. (2015). Sodium fluoride affects DNA methylation of imprinted genes in mouse early embryos. *Cytogenet. Genome Res.* 147 41–47. 10.1159/000442067 26633825

[B234] Zheng H. Y. Shi X. Y. Wu F. R. Wu Y. Q. Wang L. L. Chen S. L. (2011). Assisted reproductive technologies do not increase risk of abnormal methylation of PEG1/MEST in human early pregnancy loss. *Fertil. Steril.* 96 84–89. 10.1016/j.fertnstert.2011.04.021 21575949

[B235] Zhu W. Du J. Chen Q. Zhang Z. Wu B. Xu J. (2019). Association of UHRF1 gene polymorphisms with oligospermia in Chinese males. *J. Assist. Reprod. Genet.* 36 2563–2573. 10.1007/s10815-019-01614-7 31802345 PMC6911149

